# Adrenal causes of endocrine hypertension in childhood or adolescence

**DOI:** 10.1007/s40618-025-02633-1

**Published:** 2025-06-20

**Authors:** Bianca Pellegrini, Ilaria Bonaventura, Valeria Hasenmajer, Chiara Simeoli, Claudia Pivonello, Davide Ferrari, Sabrina Criscuolo, Alessandra Tomaselli, Andrea M. Isidori, Ashley B. Grossman, Andrea Lenzi, Maria Cristina De Martino, Martin O. Savage

**Affiliations:** 1 Dipartimento di Medicina Clinica e Chirurgia, Sezione di Endocrinologia, Andrologia e Nutrizione, Università Federico II di Napoli, Diabetologia, Naples, Italy; 2 Dipartimento di Farmacia e Scienze della Salute e della Nutrizione, Università della Calabria, Rende, Italy; 3 Department of Experimental Medicine, Sapienza University of Rome, Rome, Italy; 4 Dipartimento di Sanità Pubblica, Università degli Studi di Napoli Federico II, Naples, Italy; 5 Pediatric University Department, Bambino Gesù Children Hospital, Rome, Italy; 6 Pediatric Unit, Maternal and Child Health Department, Azienda Ospedaliera Universitaria San Giovanni di Dio e Ruggi d’Aragona, Salerno, Italy; 7 Centre for Rare Diseases (Endo-ERN Accredited), Policlinico Umberto I, Rome, Italy; 8 Centre for Endocrinology, William Harvey Research Institute, Barts and the London School of Medicine and Dentistry, Queen Mary University of London, Charterhouse Square, London, EC1M 6BQ UK; 9 NET Ubit, ENETS Centre of Excellence, Royal Free Hospital, London, NW3 2QG UK

**Keywords:** Endocrine hypertension, Congenital adrenal hyperplasia, Cushing syndrome, Phaeochromocytoma, Paraganglioma, Paediatric

## Abstract

Arterial hypertension is characterised by elevated blood pressure (BP) leading to cardiovascular morbidity and mortality, and organ damage. Its prevalence in childhood is around 5% and children should be screened from 3 years of age. Hypertension in childhood or adolescence requires exclusion of a secondary cause. Adrenal disorders frequently underlie secondary hypertension. presenting with imbalances of BP and pleiotropic clinical presentations. Examples are rare genetic defects leading to increased mineralocorticoid activity such as Congenital Adrenal Hyperplasia (CAH) due to 11β-hydroxylase gene (*CYP11B1*) or 17-hydroxylase gene *(CYP17A1)* mutation, and Familial Hyperaldosteronism (FH), due to 11β-hydroxylase 1 (*CYP11B1*) and 11β-hydroxylase 2 (*CYP11B2*) gene fusion, or to mutations of other genes involved in aldosterone production such as those codifying the chloride-voltage gated channel 2 (*CLCN2*), the potassium inwardly rectifying channel subfamily J member 5 (*KCNJ5*), and the calcium voltage-gated channel subunitsα1 H and D (CACNA1H and CACNA1D). The differential diagnosis of childhood hypertension also includes endogenous hypercortisolism (Cushing’s syndrome) or phaeochromocytomas/paragangliomas, neoplastic conditions potentially caused by germinal genetic alterations, in a specific familial syndrome. Lastly, peripheral glucocorticoid and mineralocorticoid pathway disorders due to germline mutations in *HSD-11B2*, codifying the enzyme 11β-dehydrogenase type 2, *NR3C1* and *NR3C2* genes codifying the nuclear receptor subfamily 3 group C members 1 and 2 may also be responsible. A systematic diagnostic approach based on published guidelines is still lacking, and diagnostic suspicions with referral for gene sequencing need to be identified. This review discusses the known causes of endocrine hypertension in children and adolescents, with an emphasis on prevalence, clinical presentation, genetic predisposition and therapeutic strategies.

## Introduction

Arterial hypertension is a widespread, chronic condition and represents a primary contributor to the global burden of non-communicable diseases, leading to cardiovascular morbidity and mortality, renal disease, and damage to several other organs and tissues [1]. While standardised BP ranges are commonly employed to define hypertension in adults, BP displays a trend to progressive increase with age and body size during childhood and the transition age, complicating the establishment of singular cut-offs for diagnosis of hypertension in this age range. According to the *European Society of Hypertension*, hypertension is diagnosed in children and adolescents aged 3–15 years when BP levels exceed the 95th percentile of the normative BP distribution for age, sex and height percentiles across at least three different measurements [2–4]. From 16 years of age, diagnostic criteria align with those for adults, with a cutoff of 140 mmHg for systolic BP and 90 mmHg for diastolic BP [3]. Consequently, the expected prevalence of hypertension in childhood is approximately 5%.

However, these diagnostic criteria are inconsistently applied across different countries and the normative data are somewhat outdated, making it challenging to accurately determine the prevalence of hypertension in childhood. In the American continent, the estimates range from 0.3 to 4.5% [5], but the prevalence vertiginously increases in the presence of concomitant overweight or obesity [6]. European studies report a prevalence ranging from 2 to 13% in children and adolescents [7, 8]. An Italian study evaluated 415 Italian children and adolescents referred for obesity, and found that 23.6% showed elevated BP levels [9]. The development of hypertension in childhood and adolescence is linked to an increased risk of adult age hypertension [10]. Therefore, it is recommended that BP be screened in children starting from 3 years of age for early exclusion of hypertension [2].

When hypertension is identified in children and during the transition to adulthood, the search for a secondary cause should always be made. The spectrum of potential causes of hypertension at these ages is broad, encompassing conditions such as coarctation of the aorta, renal parenchymal diseases and nephron-vascular hypertension, and other more rare causes including monogenic disorders, systemic arteritis and neurological diseases [2].

Endocrine disorders frequently underlie secondary hypertension. Indeed, the mechanism regulating BP levels are intricately intertwined with the endocrine system at various levels [11]. Notably, the adrenal glands play a pivotal role in BP regulation. Adrenocortical steroids, especially glucocorticoids and mineralocorticoids, act as BP regulators through receptor-mediated signalling pathways in the kidneys, cardiovascular system and several other organs and tissues [12, 13]. Similarly, catecholamines from the adrenal medulla target adrenergic and dopamine receptors, influencing BP both peripherally and centrally by acting on arterial smooth muscle and myocardial tissue [14]. Given the adrenal glands’ central role in BP regulation, it is not surprising that most of the adrenal disorders present with an imbalance in BP.

In this review, our investigation will focus on the characteristics encountered in diagnosing hypertension of adrenal origin during childhood and the transition to adulthood, considering variations in prevalence, clinical presentation, genetic predisposition, and therapeutic strategies.

## Rare forms of congenital adrenal hyperplasia causing

Adrenal steroidogenesis relies on enzymatic reactions leading to the synthesis of the three main groups of adrenal hormones: glucocorticoids, mineralocorticoids and androgens. Enzymatic defects throughout the pathway characterise different forms of congenital adrenal hyperplasia (CAH). They can lead to an absolute or relative deficiency of one or more hormones, and the lack of negative feedback control of pituitary adrenocorticotropic hormone (ACTH) secretion results in increased precursors that impact the clinical presentation. Hypertension due to accumulating mineralocorticoid precursors is characteristic of 11β-hydroxylase (*CYP11B1*) and 17-hydroxylase (*CYP17A1*) deficiency [15].

### 11β-Hydroxylase deficiency

#### *Diagnosis*

The identification of 11β-hydroxylase deficiency as a cause of congenital hypertensive hyperandrogenism was first proposed in the 1950 s [16], and accounts for approximately 5% of CAH cases [15]; 11β-hydroxylase is encoded by *CYP11B1* on chromosome 8q21 and more than 100 mutations have been described, including chimeric *CYP11B1/CYP11B2* forms [17] and uniparental disomy [18], but there appears to be little correlation between genotype and phenotype [19].

Defective 11β-hydroxylase activity due to mutations in the *CYP11B1* gene affects the final step of cortisol secretion, leading to accumulation of 11-deoxycortisol and other precursors in the zona fasciculata, while in the zona glomerulosa, the conversion from 11-deoxycorticosterone (DOC) to corticosterone, an aldosterone precursor, is impaired. The accumulating precursors cause clinical and biochemical hyperandrogenism and the increased DOC levels lead to hyporeninaemic hypertension. Cortisol deficiency impairs the physiological pituitary and hypothalamic feedback control, resulting in excess unregulated ACTH secretion [20].

#### Clinical presentation

The classical form of the condition are characterised by variable degrees of hypokalaemic hypertension and hyperandrogenism, resulting in virilisation in 46XX patients and increased androgenisation in 46XY patients, together with accelerated linear growth and peripheral premature puberty. Approximately two thirds of patients develop hypertension, often during childhood [21], or even at birth in one case report [22]. In a case series, a correlation between age of diagnosis and severity of hypertension is reported [23]. Hypertension is usually of mild-to-moderate severity, despite a few cases of malignant hypertension [24, 25]even requiring salvage adrenalectomy [24]. Accordingly, left ventricular hypertrophy, retinopathy, hypertensive nephropathy, ischaemic heart disease, cerebrovascular accidents, and deaths are reported [24–27] The age of diagnosis is very variable and often sex-related, since genital ambiguity in females is detected at birth and leads to earlier evaluations compared to males. In the largest studied cohort from a multicentre study, age at diagnosis ranged between birth and 17 years [28]. In untreated males, testicular adrenal rest tumours (TART) may develop [29–32].

Non-classical forms of CAH due to 11β-hydroxylase deficiency have also been described [33]^,^ mimicking polycystic ovary syndrome and representing a potentially understudied phenotype of this rare condition.

#### Treatment

Management includes appropriate glucocorticoid replacement as required, along with additional anti-hypertensive therapy if needed [34]. Preferred therapies are Mineralocorticoid Receptor (MR) Antagonists (MRAs), such as spironolactone and eplerenone, and epithelial sodium channel (ENaC) blockers such as amiloride [2]. For the treatment of androgen excess, glucocorticoid replacement is usually able to at least partially normalise levels of androgen precursors; however, other therapeutic strategies, such as anti-androgen therapies, might be required in severe virilisation. Synthetic long-acting steroids are not routinely recommended to achieve ACTH suppression. Adrenalectomy, on the other hand, remains an option in uncontrolled cases.

### 17-Hydroxylase/17,20-lyase deficiency

#### Diagnosis

The first case of 17-hydroxylase deficiency was reported in 1966 [35], describing a 46XX woman, aged 35 years old, with hypertension, prepubertal breast tissue and lack of axillary and pubic hair: 17-hydroxylase/17,20-lyase deficiency is rare, accounting for approximately 1% of CAH. Moreover, complete 17-hydroxylase/17,20-lyase deficiency and the much rarer isolated 17,20-lyase deficiency have been described as separate conditions [36]. Overall, 17-hydroxylase/17,20-lyase deficiency is more common in certain regions such as Brazil [37, 38] Japan [39] and China [40]^,^ and more than 90 mutations have been identified.

The human *CYP17A1* gene is located on chromosome 10q24.3 and is transcribed both in the adrenals and gonads. It catalyses both 17-hydroxylase and 17,20-lyase activities, allowing transformation of pregnenolone and progesterone into 17-OH-pregnenolone and 17-OH-progesterone, respectively, while 17,20-lyase is needed to produce C19 sex steroids [41]. Due to the description of a few cases of isolated 17,20-lyase deficiency with preserved 17-hydroxylase function, the two activities were assumed to be related to two different enzymes, but further studies have identified P45017a as a single polypeptide catalysing both reactions [42]. Isolated 17,20-lyase has been attributed to mutations in the binding regions of *P45017α* with cytochrome P450 oxidoreductase and microsomal cytochrome b5. These cofactors are considered “redox partners” for P45017α, and are needed to catalyse the 17,20-lyase reaction. Mutations of the *POR* gene encoding for cytochrome P450 oxidoreductase, affecting its binding to P45017α, have been described, and are phenocopies of *CYP17A1* mutations causing isolated 17,20-lyase deficiency [43, 44].

Thus, defective *CYP17A1* activity leads to increased mineralocorticoid precursors, while the cortisol and sex steroid pathways are blocked, the latter in the gonads as well. The overproduction of DOC leads to hypokalaemic hypertension in this condition. Due to the increased levels of corticosterone, glucocorticoid activity is usually preserved, but with altered ACTH feedback.

#### Clinical presentation

Diagnosis is often delayed due to the lack of adrenal insufficiency and an elusive clinical picture during childhood. In fact, the profound androgen and oestrogen deficiency due to altered 17,20-lyase activity leads to abnormal sexual differentiation with external female genitalia in 46XY individuals and a lack of breast development and primary amenorrhoea in 46XX individuals, even though the phenotypic spectrum is rather broad, as described by several case series [45]. Accordingly, most patients are diagnosed during adolescence [46].

In 46XX patients, ovarian cysts, amenorrhoea and infertility are common, due to persistently high progesterone levels, increased FSH and LH and low oestrogen [47]. Ovulation induction and successful pregnancies through medically-assisted reproduction techniques have been described in patients with complete and incomplete 17-hydroxylase/17,20-lyase deficiency [47, 48]. On the other hand, reports on fertility preservation in 46XY males with this condition have not been published. Hypertension in 17-hydroxylase/17,20-lyase deficiency, similarly to 11β-hydroxylase deficiency, is due to salt retention and volume expansion caused by excess mineralocorticoid precursors, and is usually mild. Due to the delay in diagnosis, hypertension is most commonly described during adolescence [49]. Severe hypertension can be an unusual presenting symptom, leading to earlier diagnosis, as described in a case series of 12 patients [46]. Malignant hypertension, even leading to encephalopathy and death, has also been described [46, 50].

#### Treatment

Glucocorticoid replacement is usually not required due to high corticosterone production and might lead to hypothalamo-pituitary-adrenal (HPA) axis suppression with increased risk for adrenal crises. MRAs are usually successful in achieving target BP levels [51] and, due to lack of virilisation, side effects in the 46XY population such as gynaecomastia and erectile dysfunction are usually absent. Hormonal replacement therapy is usually required in 46XX patients, along with testosterone therapy for 46XY patients, reared or reassigned as males by patient and family preference.

## **Familial hyperaldosteronism (FH**)

Primary Aldosteronism (PA) is the most common cause of secondary hypertension in the paediatric and transitional age. In approximately 5% of cases, it is hereditary and is referred to as familial hyperaldosteronism (FH) [52]. Within the definition of FH, some disorders present with heterogeneous clinical features and age at diagnosis ranging from the first months of life to the transitional age, indeed the current guidelines establish < 20 years as the cut-off age for clinical suspicion of FH [53]. In addition to type 1 FH or glucocorticoid-remediable aldosteronism, three other forms of FH are currently known.

FH1 is caused by a chimeric 11β-hydroxylase 1 (*CYP11B1*)−11β-hydroxylase 2 (*CYP11B2*) gene, which leads to ACTH-stimulated synthesis of aldosterone. The other types of FH are due to altered function of anion or cation channels which are involved in the regulation of aldosterone production. In-depth study of the molecular alterations underlying FH, their pathophysiological implications, and the clinical characterisation of affected families, has allowed precise predictions of genotype-phenotype correlations, although much remains to be understood about the pathogenesis and clinical manifestations of FH [54].

### FH1

#### Pathogenesis and diagnosis

FH1, the first monogenic form of FH identified, is also known as glucocorticoid-remediable aldosteronism [55] and accounts for 1% of PA cases and 3.1% of cases of hypertension onset between ages 4 and 15 [55, 56]. Inherited as an autosomal dominant disorder, it is caused by an unequal crossover between the *CYP11B1* and *CYP11B2* genes, which are 95% homologous, on chromosome 8 (locus 8q24). The resulting chimeric gene contains the promoter of *CYP11B1* and the coding portion of *CYP11B2*. In the zona fasciculata of the adrenal cortex, this results in abnormal activity of 11β-hydroxylase-aldosterone synthase under non-physiological ACTH stimulation [57, 58]. Although a wild-type copy of the aldosterone synthase gene is present, aldosterone production is not responsive to angiotensin stimulation so the hybrid gene’s function predominates over the persistently deficient wild-type gene [59, 60]. Furthermore, ACTH stimulation activates cortisol C-18-hydroxylation and C-18-oxidation by aldosterone synthase, resulting in the over-secretion of 18OH-cortisol and 18-oxo-cortisol [61].

#### Clinical presentation

The clinical presentation of FH1 is highly variable, being generally characterised by arterial hypertension before the age of 20 (mean age 18 ± 17.6 years) [62] and rarely accompanied in early childhood by cerebellar infarction or intracranial haemorrhage [55, 62].A recent systematic review revealed that among individuals with a confirmed genetic diagnosis of FH1, about 77% were hypertensive and about 42% were hypokalaemic [62].

Hypertension in FH1 is often resistant to polypharmacotherapy and accompanied by early micro/macrovascular complications, such as vascular dissections (sometimes as early as age 10), retinopathy (mean age 16.5 years), nephropathy, cardiac remodelling with left ventricular hypertrophy (mean age 15 years), and increased risk of acute myocardial infarction and stroke at a young age (before age 40 years) [62, 63]. Males tend to present with a more severe phenotype and worse long-term cardiovascular prognosis compared to females [64].

A good clinical response to glucocorticoid administration strongly suggests the diagnosis of FH1. To confirm the diagnosis, dexamethasone suppression tests for aldosterone and measurements of hybrid steroids, particularly 18-oxo-cortisol, have been suggested. The levels of 18-oxo-cortisol seem to correlate with the “mean day curve control” achieved with medical therapy [60, 65]. However, studies on large populations of FH1 patients have shown that the aldosterone-renin ratio (ARR) remains probably the more reliable biochemical marker of the disease, with no significant differences in biochemical parameters between subjects with mild and severe phenotypes. Definitive diagnostic confirmation requires molecular analysis to detect the chimeric gene [64].

However, different possible locations of the crossover breakpoints have been described and probably result in considerable genotypic variability. Indeed, some studies have shown that the hybrid gene, considered pathognomonic, can be detected only in a minority of FH1 patients, and that the use of plasmids containing a large fragment of the wild-type *CYP11B2* gene and the chimeric gene - including segments where the most frequent breakpoints occur - could represent more sensitive tools for the molecular diagnosis of FH1 [66, 67]. The adrenal morphological findings on imaging are variable and include normal adrenal glands, bilateral adrenal hyperplasia, and adrenal adenomas [56].

#### Treatment

Current guidelines recommend a low-dose, long-acting glucocorticoid taken before bedtime to suppress ACTH, with the addition of MRAs such as spironolactone if no satisfactory clinical response is achieved. The goal of medical therapy is to adequately balance the benefit of controlling BP and electrolytes with the risk of excessive corticosteroid exposure and, in the case of some MRAs, undesirable effects such as gynaecomastia in males. Overall, safer partial suppression of ACTH might be sufficient for disease control [62].

### FH2

#### Pathogenesis and diagnosis

Initially, all non-FH1 cases of FH were described as FH2 [58]. This broad definition included a large number of heterogeneous cases from chemical, biochemical, and radiological perspectives, sometimes indistinguishable from sporadic PA [68]. At the same time, it entailed a significant risk of overestimating the prevalence of FH2. Indeed, considering the high prevalence of PA in the general population there is a non-negligible probability that at least two cases of sporadic PA could coexist in the same family [56, 69]. In recent decades, gene sequencing has enabled the identification of genes whose mutations are responsible for different types of FH. Currently, experts suggest that the definition of FH2 should refer only to forms due to germline mutations of *CLCN2*, codifying the chloride-voltage gated channel 2 (ClC2). FH2 represents around 5% of PA cases.

*CLCN2* is a highly conserved gene among different species, located on the long arm of chromosome 3 (locus 3q27.1) [56]. ClC2 is expressed in various human organs and tissues, including the brain, intestine, lung, and adrenal cortex [56]. Immunohistochemical studies have demonstrated a predominant localization of ClC2 in the zona glomerulosa of the adrenal cortex [70]. Moreover, somatic mutations of *CLCN2* have been observed in aldosterone-producing adenomas in association with more severe biochemical hyperaldosteronism, thus suggesting the important role of ClC2 in determining aldosterone synthesis [54, 71, 72].

The ClC2 channel is a homodimer, each subunit containing a conduction pore. Gate opening can occur with a rapid activation of individual subunits or with a slow combined activation of the two subunits. ClC2 undergoes slow activation at potentials more negative than the chloride equilibrium potential. At the resting potential of zona glomerulosa cells, ClC2 is slowly activated. The increased sodium efflux leads to a faster cell depolarisation, followed by the activation of voltage-dependent calcium channels, which results in the overexpression of genes involved in aldosterone synthesis, including *CYP11B2* [70, 73].

Currently, six mutated variants of ClC2 have been identified in patients with FH2:

- *pSer865Arg*: at the C-terminal end; modifies channel gating, especially with increased fast gate opening.
- *pArg175Gln*: at the cytosolic end of the D-helix; through interaction with the C-terminal end, alters anionic selectivity and gating.
- *pLys263de*l: in-frame deletion at the loop between helices J and K; facilitates gate opening.
- *pGly24Asp*,* pMet22Lys*,* pTyr26Asn*: at the N-terminal end in the inactivation domain; causes greater current flows at the resting potential [74].

Biochemical investigations show elevated aldosterone, suppressed renin, and hypokalaemia. Given the absence of pathognomonic clinical features or specific biochemical markers of FH2, definitive diagnostic confirmation requires molecular investigations identifying germline mutations of *CLCN2* [69].

#### Clinical presentation

The clinical presentation of FH2 is variable and may include hypertension with onset at a young age, often before 20 years, and sometimes hypokalaemia [75]. As in FH1, autosomal dominant inheritance with incomplete penetrance has been hypothesised, as many individuals are heterozygous and some cases of patients with a normal aldosterone/renin ratio or spontaneous improvement of hypertension with increasing age have been observed [56]. Adrenal morphology is variable (normal glands, adenomas, bilateral hyperplasia).

#### Treatment

Generally, a good clinical response, with improvement of hypertension and hypokalaemia when present. is achieved with medical therapy using MRAs as spironolactone or amiloride [56, 69].

### FH3

#### Pathogenesis and diagnosis

FH3 is defined as FH due to germline mutations of potassium inwardly rectifying channel subfamily J member 5 gene (*KCNJ5*), which is estimated to account for 8% of FH cases and 0.6% of PA cases [56]. The *KCNJ5* gene is located on the long arm of chromosome 11 (Locus 11q24.3) and encodes the potassium channel KIR3.4 [76]. Therefore, FH3 can be considered a veritable channelopathy [77].

In humans, KIR3.4 is expressed in various organs and tissues, including the heart, central and peripheral nervous system, and adrenal cortex [78]. Immunohistochemical studies on the adrenal cortex have demonstrated predominant localisation of *KIR3.4* in the zona glomerulosa [79]. Next-generation sequencing analyses have identified somatic mutations of *KCNJ5* in about 40% of aldosterone-producing adrenocortical adenomas, associated with more severe hyperaldosteronism, suggesting a direct role of *KCNJ5* in determining aldosterone overproduction and proliferation of the zona glomerulosa cells [58, 80].

*KIR3.4* can assemble in homotetramers or heterotetramers with *KIR3.1* [81]. At the resting potential of the zona glomerulosa cells of the adrenal cortex, *KIR3.4* helps to maintain membrane hyperpolarisation, thanks to high conductance potassium efflux [76]. Mutations in *KCNJ5* responsible for FH3 cause reduced channel selectivity, resulting in increased sodium influx and membrane depolarisation, followed by the activation of voltage-dependent calcium channels, increased intracellular calcium, and the upregulation of genes that contribute to aldosterone synthesis [82].

Currently, 9 mutated variants of *KIR3.4* have been identified in patients with FH3:

- *Thr158Ala*,* Gly151Arg*,* Ile157Ser*,* Gly151Glu*,* Glu145Gln*,* Tyr152Cys*,* pThr149Del*, with substitutions or deletions of amino acids in or near the selectivity filter.
- *Val259Met* and *Tyr348Asn* near the C-terminal cytoplasmic end [78, 79, 83, 84].

#### Clinical presentation

Considerable clinical heterogeneity has been documented among families affected by FH3. A classification of FH3 into two subtypes has been proposed: the more severe type A and the milder type B [85]. Furthermore, specific genotypes underlying the milder/more severe phenotypes have also been suggested. Indeed, some *KCNJ5* mutations cause more marked alterations in *KIR3.4* permeability to sodium and may be potentially lethal for zona glomerulosa cells [58]. The latter mutations are associated with clinical presentations characterised by less severe hyperaldosteronism and the absence of marked adrenal hyperplasia [82].

A more severe clinical presentation with young age-onset hypertension resistant to pharmacological treatment, hypokalaemia, polyuria, nocturia, polydipsia, myalgia, and in one case, growth retardation, has been described in patients with mutated variant *Thr158Ala*,* Gly151Arg*,* Glu145Gln* or *Ile157Ser* [78, 86–88]. In these cases the imaging of the abdomen shows massive bilateral adrenal hyperplasia, often with a macronodular aspect. Histological examination reveals a loss of zonation and difficulty distinguishing zona glomerulosa cells, confirmed by co-expression of *CYP17*,* CYP11B1*, and *CYP11B2* in some cells on immunohistochemistry [89].

Milder clinical presentations have been observed in patients with the variants of *Gly151Glu* and *Tyr152Cys*, presenting with severe hypertension at a young age and hypokalaemia showing favourable disease progression during growth, and later-onset hypertension in some cases. Radiological findings do not show significant adrenal hyperplasia, except in one case of germline mosaicism of *KCNJ* with bilateral adrenal hyperplasia [90]. Good clinical and biochemical control is achieved with pharmacological therapy: MRAs such as spironolactone or canrenone and other anti-hypertensives such as ACE inhibitors or β-blockers. verapamil or amiloride [78, 91].

Patients with *Val259Met* and *Tyr348Asn* variants present with hypertension after age 50, normal aldosterone-renin ratio values, but ACTH-stimulated aldosterone hypersecretion, in the presence of normal adrenal glands on abdominal imaging [92]. The only one patient with *pThr149Del* variant developed hypertension at age 11 complicated by organ damage, alkalosis, and hypokalaemia, which was well controlled with medical therapy. Adrenal MRI showed unilateral pseudo-nodular thickening [76].

#### Treatment

Medical treatment with MRAs is generally described as the first treatment in children or adolescents affected by FH3, eventually in combination with β-blockers, ACE-inhibitors or other anti-hypertensive drugs if BP is not adequately controlled [78]. In most severe cases, when hypertension and hypokalaemia are resistant to medical treatment, adrenalectomy may be required to achieve clinical control [69]. 

### FH4

#### Pathogenesis and diagnosis

FH4 is defined as FH due to germline mutations of the calcium voltage-gated channel subunits α 1 H gene (*CACNA1H)* and is inherited as an autosomal dominant disorder [93, 94].

The *CACNA1H* gene is located on the short arm of chromosome 16 (Locus 16p13) and encodes the α subunit of the T-type voltage-dependent calcium channel Cav3.2 [56]. *CACNA1H* is widely expressed in the zona glomerulosa of the adrenal cortex, where it is involved in the regulation of *CYP11B2* expression and aldosterone production in response to membrane potential fluctuations, including the nervous system. Indeed, independently of hyperaldosteronism, *CACNA1H* variants have already been associated with absence epilepsy and idiopathic generalised epilepsy [58].

In vitro studies have demonstrated that gain-of-function variants of *CACNA1H*, when stimulated by potassium, lead to over-expression of *CYP11B2*. The prevalence of somatic *CACNA1H* mutations in aldosterone-producing adrenocortical adenomas is 4%, and it is unclear whether these mutations play a role in cell proliferation in aldosterone-producing adrenocortical adenomas besides determining the hormonal hypersecretion [58, 95, 96].

Currently, five variants of Cav3.2 due to mutations in highly conserved regions of *CACNA1H* and responsible for FH4 are known:

- *Met1549Val*: in a portion of the transmembrane segment S6 of repeat domain 3, leading to channel activation with calcium influx at less negative potentials and slow inactivation [97].
- *Ser196Leu*: in the voltage sensor region, in the transmembrane segment S4 of repeat domain 1.
- *Pro2083Leu*: in the C-terminal cytoplasmic domain.
- *Val1951Glu* and *Met1549Ile*: in the C-terminal cytoplasmic domain, likely involved in the channel activation [98].

Despite autosomal dominant inheritance, cases of subjects carrying *CACNA1H* mutations with no hypertension even in adult age, or without biochemical evidence of hyperaldosteronism, have been described. Incomplete penetrance due to genetic mosaicisms or a tendency for the clinical picture to ameliorate with increasing age could be suggested [58].

#### Clinical presentation

The clinical presentation typically associated with the *Met1549Val* mutation includes the onset of hypertension before the age of 10 years, without a history of epilepsy or neuropsychiatric disorders, while patients with the *Met1549Ile* variant have shown mental retardation and learning disorders [97, 98]. In one patient with the *Met1549Val* mutation, growth retardation has been described [99].

Interestingly, a *de novo* missense germline mutation in the transmembrane voltage sensor domain was described in a 31-year-old patient with PA and no family history of hypertension or hypokalaemia. This mutation caused a loss of function of Cav3.2 and reduced whole-cell current, indicating that the pathogenic role of *CACNA1*H is still not fully understood [99].

Radiological findings show normal adrenal glands in the majority of cases, particularly in association with the *Met1549Val* and *Met1549Ile* variants. Gland thickening without nodules, single or bilateral adrenal nodules, are associated with the variants *Pro2083Leu*,* Val1951Glu*, and *Ser196Leu*, respectively [94].

#### Treatment

The goal of FH4 therapy is clinical control of hyperaldosteronism, generally achieved with MRAs and, if necessary in the presence of aldosterone-producing adrenocortical adenomas, adrenalectomy.

## CACNA1D-related hyperaldosteronism

### Pathogenesis and diagnosis

In addition to the monogenic forms of PA described above, germline mutations of the calcium voltage-gated channel subunits α 1 D gene (*CACNA1D)* can cause PA in early childhood. The *CACNA1D* gene, located on the short arm of chromosome 3 (Locus 3p14.3), encodes the pore-forming α1 subunit of the L-Type voltage-dependent calcium channel Cav1.3 [58, 100]. In humans, Cav1.3 is expressed in the zona glomerulosa of the adrenal cortex, in the brain, in pancreatic β cells, in the sinoatrial node, and in cochlear hair cells [101–103].

### Clinical presentation

Homozygous mutations in *CACNA1D* lead to severe cardiac conduction disorders and congenital deafness, while heterozygous mutations can lead to various clinical presentations, including neuropsychiatric disorders, neonatal hyperinsulinaemic hypoglycaemia, and PA [102, 103].

Somatic mutations of *CACNA1D* are among the most frequent mutations in aldosterone-producing adrenocortical adenomas, particularly in the absence of *KCNJ5* mutations, where the gain of function of Cav1.3 is responsible for aldosterone production [104, 105]. Germline mutations of *CACNA1D*, some of which are identical to somatic mutations in aldosterone-producing adrenocortical adenomas, cause PA with epilepsy and neurological abnormalities in early infancy (the so-called primary hyperaldosteronism-seizures-neurological abnormalities syndrome) [106].

Currently, 4 mutated variants of *Cav1.3* responsible for childhood PA are known:

- *Gly403Asp*.
- *Ile770Met*.
- *Phe767Leu*.
- *Thr776Ala*.

These mutations involve domains responsible for channel opening, in highly conserved regions of Cav1.3, and channel activation at less depolarised potentials and interfere with channel inactivation [106].

The clinical presentation includes neurological disorders (epilepsy, cerebral palsy, cerebral blindness etc.), cardiac abnormalities (ventricular hypertrophy, atrial septal defects), and PA with arterial hypertension and hypokalaemia. Hypertension onset has been described at 1–3 months of life associated with the *Gly403Asp* and *Thr776Ala* variants, and at age 5 associated with the *Ile770Met* variant [104, 107].Recently, a case of *de novo* mosaicism of the *Phe767Leu* variant was described in a patient with Chiari malformation, chorea, developmental delay, neonatal hypoglycaemia, and low renin levels [102].

#### Treatment

As demonstrated by preclinical studies and clinical experience, given the central pathogenic role of altered calcium signalling in PASNA syndrome, good control of both PA and neurological symptoms can be achieved with dihydropyridine calcium antagonists [104, 108].

## Paediatric cushing’s syndrome

### Pathogenesis

Cushing’s syndrome (CS) is caused by prolonged exposure to elevated circulating levels of cortisol [109–112]. The majority of cases, 70–80%, of endogenous CS are due to hypersecretion of ACTH by the pituitary gland at all ages [109–114]. This excessive ACTH production stimulates the adrenal glands to overproduce cortisol, a condition known as pituitary-dependent CS or Cushing’s disease (CD). On the other hand, ACTH-independent adrenal production of cortisol by an adrenocortical adenoma, carcinoma or rare forms of bilateral adrenal disease, accounts for the remaining percentage of cases of endogenous CS [109–112].

However, in children aged less than 5 years, CD is extremely rare and the most common cause of CS in this age group is ACTH-independent CS (due to adrenocortical adenoma, carcinoma or bilateral adrenal hyperplasia) [113, 114]. Lastly, although extremely rare in the paediatric age range, an extra-pituitary tumour that secretes ACTH or, even more rarely, corticotropin-releasing hormone (CRH), causes ectopic CS [115]. Endogenous CS is rare in children and adolescents, compared to its frequency in adults. The annual incidence in the general population is estimated to be between 0.7 and 2.4 cases per million individuals, and only 10% of new cases occur in children each year [113, 114]. In prepubertal paediatric patients, CD is commoner in boys than in girls, with the sex incidence equalising during puberty and then assuming the adult female dominant pattern after puberty [113, 114, 116].

Albeit rare, several genetic mutations are responsible for syndromes associated with paediatric CS [117, 118]. McCune-Albright syndrome results from somatic mutations of the *GNAS* gene, specifically mutations in the cAMP-regulating protein, Gsα, that is constitutively activated [117, 119]. McCune-Albright syndrome is characterised by the clinical triad of fibrous dysplasia, café-au-lait skin pigmentation, and precocious puberty, and may include other endocrine abnormalities, such as CS, excess growth hormone secretion, and hyperthyroidism [119]. In McCune-Albright syndrome, the adrenal glands develop a unique form of adrenal hyperplasia, termed primary bimorphic adrenocortical disease, characterised by multiple nodules arising from adrenocortical cells with fetal characteristics [120].

Primary pigmented nodular adrenocortical disease (PPNAD) is a genetic disorder usually associated with Carney complex, a syndrome of multiple endocrine gland abnormalities caused by inactivating germline mutations of the *PRKAR1A* gene, leading to constitutive activation of the cAMP–PKA pathway by increasing the availability of the PKA catalytic subunits [121]. The adrenal glands in PPNAD are characterised by multiple pigmented nodules that autonomously secrete cortisol and are surrounded by an atrophic cortex [121]. Primary bilateral macronodular adrenal hyperplasia (PBMAH) is frequently a genetic disorder, most often caused by inactivating mutations of *ARMC5*, a putative tumour suppressor gene [117, 122]. Histologically, PBMAH is composed of numerous nodules measuring > 1 cm each and in more than 90% of cases PBMAH is characterised by varying degree of hypercortisolism [122–124].

Multiple endocrine neoplasia type 1 (MEN 1) is an autosomal dominant disorder due to mutations in the *MEN1* gene, which codes for the protein menin. It is characterised by neuroendocrine tumours, mainly affecting the parathyroid glands, pancreatic islet cells and anterior pituitary. Although rare, CD can occasionally be associated with MEN 1 [125].

The most prevalent somatic mutations in paediatric CD occur in the *USP8* gene, which have been detected in 31–63% of corticotroph adenomas. The *USP8* gene encodes a deubiquitinase enzyme responsible for regulating tyrosine kinase receptors, such as the epidermal growth factor receptor (EGFR) [118]. Deubiquitination of EGFR prevents its degradation in lysosomes, thereby maintaining downstream signalling pathways [126, 127]. While biochemical markers of hypercortisolism, tumour size, and the frequency of cavernous sinus invasion, show no significant differences between subjects with and without *USP8* mutations, individuals with these mutations have a higher risk of CD recurrence following transsphenoidal surgery (TSS) compared to those without mutations [126].

Childhood adrenocortical carcinomas (ACC) may be associated with Li-Fraumeni and Lynch syndromes [128]. Genetic diagnosis of Li-Fraumeni syndrome is usually made by germline analysis for variants in *TP53* [129]. Evaluation for Lynch syndrome can be initiated by immunohistochemistry for MSH2, MLH1, PMS2, MSH6 and microsatellite instability testing, or by direct germline genetic analysis for *MSH2*,* MLH1*,* PMS2*,* MSH6 and EPCAM* [130]. All children diagnosed with ACC should undergo a systematic search for germline *TP53* pathogenic variants, as 50–90% of childhood ACC is associated with such variants [128].

Hypertension is common at diagnosis (36–71%) and its severity is mainly related to the duration and intensity of elevated cortisol levels, due to the induction of the mineralocorticoid response mediated by cortisol excess [3, 131–136]. Specifically, the MR is activated by the saturation of the enzyme 11β-hydroxysteroid dehydrogenase type 2 (11β-HSD2), which converts cortisol to inactive cortisone, thus protecting it from cortisol binding [135, 136]^127^. As a result, cortisol binds to the MR, mimicking the action of aldosterone, leading to renal sodium uptake and potassium excretion, resulting in blood volume expansion and hypertension [136–138]. Moreover, glucocorticoids modulate the synthesis and the vascular response to catecholamines, resulting in an enhanced pressor response to β-adrenergic agonists and impaired cardiac sympathetic autonomic modulation [136].

In addition, hypercortisolism enhances the vascular response to vasoconstrictors, leading to increased BP levels [136]^126^. Additionally, cortisol exerts an indirect effect on BP and the vasculature through the systemic complications of CS, such as the metabolic syndrome, and obstructive sleep apnoea [132, 139]. Typically, the loss of physiological nocturnal BP decreases to the extent that non-dipping hypertension is one of the main cardiovascular manifestations in patients with CS, reflecting the impairment of circadian cortisol secretion [132, 139]. Given the high prevalence of hypertension in these patients, an early diagnosis of CS is advisable in order to reduce the cardiovascular complications to which these patients are predisposed [3, 131].

#### Diagnosis

In paediatric patients with suspected CS, exclusion of exposure to exogenous glucocorticoids prior to biochemical evaluation is mandatory. After an initial evaluation that includes assessment of auxological parameters, pubertal stage, virilisation and bone age, documentation of hypercortisolism is crucial, usually using 24-hour urinary free cortisol (UFC), late night salivary cortisol (LNSC) or sleeping midnight serum cortisol determination, and dexamethasone suppression testing (DST). Due to imperfect diagnostic accuracy and limitations, multiple tests are often required to confirm hypercortisolism [140, 141]. Once hypercortisolism has been diagnosed, it is important to differentiate ACTH-dependent from ACTH-independent CS using hormonal assays and imaging. CD typically shows detectable morning plasma ACTH, whereas primary adrenal disease shows suppressed levels (ACTH < 5 pg/mL) [141]. In the latter case, imaging of the adrenal glands is indicated. The CRH stimulation test may show an exaggerated cortisol response in pituitary adenomas, suggesting a diagnosis of CD [142], although due to a lack of availability desmopressin may be used instead. Pituitary Magnetic Resonance Imaging (MRI) assists in this diagnosis but has limited accuracy, revealing an abnormal pituitary image in approximately 60% of cases of CD. Bilateral inferior petrosal sinus sampling (BIPSS) is recommended along with CRH or desmopressin stimulation in paediatric cases with a negative MRI and confirmed hypercortisolism to rule out ectopic [143].

#### Clinical presentation

Key diagnostic features in children with CS, are facial rounding, weight gain, growth retardation and increased virilisation [113, 114]. Facial changes occur gradually and are often initially unnoticed, while weight gain is more rapidly noticed by relatives [144]. Growth retardation does not always lead to short stature, but growth velocity is reduced [145]. Virilisation manifests as advanced Tanner stage pubic hair growth compared with breast development or testicular volume, although this can be difficult to detect in pubertal patients [144].

Other less specific symptoms include osteopenia, hirsutism, mood changes, striae, hypertension, acne, and delayed puberty [113, 114]. Despite short stature, bone age typically remains within the normal range due to increased adrenal androgen secretion [146]. Long-term hypercortisolism may lead to gonadotrophin deficiency, evidenced by decreased testicular volume or breast development along with advanced pubic hair growth [146].

#### Treatment

The primary treatment for CD in children is transsphenoidal surgical resection (TSS) of the pituitary tumour, with success rates of 70 − 100% in experienced centres [142]. However, surgery can fail, or CD can recur, with lower success rates for repeat TSS [147]. Complications after surgery include various hormonal imbalances and rare cases of mortality [148]. Hypopituitarism is a common consequence that requires frequent monitoring. Radiotherapy (RT) of the pituitary gland, commonly used in adults, is generally avoided in children due to potential complications, although it may be more effective in a shorter time-frame [149]. Innovative RT techniques such as stereotactic radiosurgery are promising, with high tumour control rates and potentially reduced side effects [150].

Medical treatment of paediatric CS is under-reported. Ketoconazole, a steroidogenesis inhibitor, is commonly used to relieve symptoms and await radiotherapy [151]. Low-dose mitotane, an adrenolytic drug used in adrenocortical carcinoma and in patients with very severe CS, showed promise but should be used with caution because of adverse events [152, 153]. Trials of osilodrostat, a 11β hydroxylase blocking agent, approved for adults, are ongoing in children [154]. Benign adrenal tumours are usually treated with surgical resection, while bilateral adrenocortical hyperplasia usually requires bilateral adrenalectomy [155]. Treatment guidelines for children with adrenal cancer are lacking because of the rarity of this condition.

In cases of ectopic CS, if the source of ACTH secretion can be identified, the treatment of choice is surgical removal of the tumour [156, 157]. If surgical resection is not feasible or the source of ACTH cannot be identified, medical therapy is indicated, as previously discussed [156, 157].

Bilateral adrenalectomy may be considered for severe hypercortisolism and life-threatening clinical morbidities per se, or be performed when more definitive treatments, such as TSS, are not possible [142]. In addition, it can be considered a therapeutic option even in rare cases of ectopic CS, when the primary tumour is occult [156, 157]. Despite a remission rate of virtually 100%, complications such as Nelson’s syndrome, defined as radiological progression or new detection of a pituitary tumour on thin-section MRI associated with hyperpigmentation and increasing ACTH levels, may occur [158, 159]. When disease remission is achieved with reduced cortisol levels, complications mediated by excess cortisol also tend to improve. However, after cortisol normalisation, systolic BP remains elevated in more than 30% at 3 months after surgery and persists in more than 5% at 12 months after surgery [134]. A significant positive correlation was observed between systolic BP and disease duration [133, 134].

## Phaeochromocytoma and catecholamine-secreting paraganglioma

The classification in 2017 of catecholamine-producing tumours from chromaffin cells of the adrenal medulla and sympathetic ganglia was developed by the World Health Organization (WHO): phaeochromocytoma (PCC) and catecholamine-secreting paraganglioma (PGL) [Data from: (Pathology and Genetics of Tumours of the Endocrine Organs. WHO Classification of Tumours, DeLellis RA, Lloyd RV, Heitz PU, Eng C (Eds), IARC press, Lyon, France 2004.)] [160]. According to the *Endocrine Society* recommendations, PCC and PGL are typically classed together and referred to as phaeochromocytomas and paragangliomas (PPGLs) [161]. The incidence of pheochromocytoma in children is approximately 1 in 1,000,000 per year, representing approximately 10% of all childhood adrenal tumours. Paragangliomas are even rarer and may be located in areas outside the adrenal glands, such as the neck, thorax, or abdomen. The majority of children are diagnosed before the age of 20, with a peak incidence in early childhood (3–5 years) for pheochromocytomas and later in adolescence for paragangliomas. PPGLs are probably seen in less than 0.2% of hypertensive adult patients [162, 163], while in children with hypertension the prevalence is higher and estimated at 1.7% [161]. Hereditary PPGLs can be divided into two cluster groups. Cluster 1 includes those due to mutations in genes encoding the von Hippel-Lindau (VHL) suppressor, the four-succinate dehydrogenase complex subunits (*SDHA*,* SDHB*,* SDHC and SDHD*) and, less commonly, the *SDHA* subunit flavinating enzyme (SDHAF2), and genes controlling fumarate hydratase, malate dehydrogenase2 and prolylhydroxylases 1 and 2 [164, 165].

The second cluster group comprises tumours caused by mutations in the neurofibromatosis type 1 (*NF1*) tumour suppressor gene, the rearranged during transfection (*RET*) proto-oncogene, the transmembrane protein 127 (*TMEM12*7) gene and the MYC-associated factor X (*MAX*) gene [166–169]. These mutations cause dysregulation of cellular metabolism and the accumulation of intermediate metabolites, oncometabolites, which can contribute to tumour formation and progression. There is third cluster, which is extremely rare, involving mutation in then Wnt signalling pathway. There is a higher prevalence of extra-adrenal, multifocal, metastatic, recurrent, and hereditary PPGLs in children than adults [170–172]. Additionally, it establishes a correlation between these phenotypic features and a higher prevalence of noradrenergic and related cluster 1 hereditary tumours in pediatric patients compared to adults. However, in young people < 18 years, the distribution of germline mutations is significantly different, with *VHL* and *SDHx* mutations relatively more common, and *MEN2* much less so [173].

### Diagnosis

Clinical suspicion of PPGL should be raised in paediatric patients presenting with (a) signs and symptoms of catecholamine excess and (b) an incidentally discovered adrenal or extra-adrenal mass. Several investigations have demonstrated that assays of metanephrines, whether in plasma or urine, are superior to those of urine vanillylmandelic acid and homovanillic acid, or serum and urine catecholamines [174, 175].

Most importantly, metanephrines are secreted continuously from the tumours rather than the intermittent bursts of catecholamines. Biochemical testing for patients with a suspected PPGL should include plasma-free or urine (spot or 24-h) levels of normetanephrine and metanephrine. The assay should be conducted using liquid chromatography [174].Generally, plasma metanephrines have the highest sensitivity and specificity, but these are closely followed by urinary metanephrines. In the paediatric and adolescent age group, sample collection may be problematic. It is usually best to have the young person quietly recumbent for 20–30 min, with an in-dwelling cannula inserted after the use of a local anaesthetic cream [176, 177]. Most urinary collections are 24 h collections, usually acceptable for teenagers but difficult in a younger age group, when a random sample corrected for creatinine may be more suitable. Nowadays, measurement is best by LC-GCMS, which is least likely to be influenced by any concomitant medication, although these should always be borne in mind. Plasma or urinary samples collected during acute illness may give false positive results, while age-appropriate reference ranges should always be used, especially in the younger patient.

Imaging is often performed when biochemical testing shows an excess of catecholamine metabolites. The first step is computed tomography scan (CT) or MRI of the pelvis and abdomen are highly sensitive to detection 0.5 mm or larger cancers.

With MRI, phaeochromocytomas tend to appear hypo- or iso-intense on T1-weighted imaging and hyper-intense on T2-weighted imaging [178]. Functional imaging should be used to rule out metastases, screen for regional extension or multifocality, and diagnose incidental lesions that are highly suspect of being PPGL but have equivocal biochemical testing [178]. Various nuclear medicine techniques can be applied for functional imaging. Functional imaging techniques, such as scintigraphy with ^123^I-labelled meta-iodobenzylguanidine [MIBG] or positron-emission tomography [PET]–CT with ^68^Ga-labe classically led [Data from: ^(^Pathology and Genetics of Tumours of the Endocrine Organs. WHO Classification of Tumours, DeLellis RA, Lloyd RV, Heitz PU, Eng C (Eds), IARC press, Lyon, France 2004.)] [163, 164, 169] tetraazacyclododecane [Data from: (Pathology and Genetics of Tumours of the Endocrine Organs. WHO Classification of Tumours, DeLellis RA, Lloyd RV, Heitz PU, Eng C (Eds), IARC press, Lyon, France 2004.)] [163, 164, 169] -tetraacetic acid–octreotate [DOTATATE] or ^18^F-labeled l-dihydroxyphenylalanine [l-DOPA], are effective in localising PPGL, but where available ^68^Ga-dotatate scanning with CT or MRI is optimal [179].

#### Clinical presentation

About 50% of patients experience symptoms, which are often paroxysmal. The clinical presentation in children is often similar to adults, but there can be some variations. The condition is typically characterised by persistent or paroxysmal hypertension [180, 181], severe headache [181, 182] that may be localised to the temporal or occipital regions, palpitations, sweating and pallor. However, the range of clinical manifestations can be wide and include abdominal pain, tremors and weight loss [180, 182]. The symptoms can also be affected by the location of the tumour. Adrenal tumours that increase adrenaline and its metabolite product, metanephrine, commonly trigger an adrenergic response. Additional adrenal lesions mostly cause a noradrenergic response by secreting noradrenaline, and normetanephrine which is produced by intracellular metabolism of noradrenaline [183]. It should be noted that the presentation of phaeochromocytoma can vary widely among individuals. Some children may be asymptomatic or present with atypical symptoms. Diagnosis can be challenging as these symptoms are not specific to phaeochromocytoma and may require a high index of suspicion, especially in children.

#### Children versus Adults

While classically most patients were diagnosed on the basis of symptoms or with resistant or paroxysmal hypertension, increasingly in adults these are being picked up as incidentally-found adrenal tumours. In children and young adults, the diagnostic algorithm is rather different, with many being diagnosed on surveillance screening, the patient having been identified as contacts of index patients with a germline mutation. Indeed, germline mutations are seen in around 80% of patients in the younger age groups, as opposed to the 25–50% in adults, above the age of 25 years. These tumours are also more likely to be paragangliomas rather than phaeochromocytomas, and multifocal, and metastatic [171].

#### Treatment

Surgical removal is the primary treatment for PPGL. The decision to proceed with surgical intervention and the selected surgical approach should be discussed at a specialist multidisciplinary team meeting. The surgical procedure should be performed by a surgeon with expertise in the surgical management of PPGLs [174].However, it is important to note that during surgery, a large amount of catecholamines may be released, leading to a ‘catecholamine storm’. This can increase the risk of hypertensive crises, cardiac arrhythmias, myocardial ischaemia, pulmonary oedema, and stroke. The management of hypertension in children with phaeochromocytoma necessitates a comprehensive approach to regulate the BP while preparing the patient for surgical excision of the tumour. To manage hypertension and avoid hypertensive crises during surgery, it is common practice to begin with α-adrenergic blockade for at least 7–10 days. Phenoxybenzamine is the preferred α-blocker for use in children, although parenteral phentolamine may also be considered [184, 185]. In order to control tachycardia and palpitations, β-blockers may be added to the treatment regimen after adequate α-blockade has been achieved. It is important to note that β-blockers should not be initiated until α-blockade has been established, as unopposed α-adrenergic stimulation could worsen hypertension. Adrenergic blockade should be combined with a high-sodium diet of 5000 mg per day and a fluid intake of 2.5 L per day. In the event of hypertensive crises, it may be necessary to promptly intervene with short-acting anti-hypertensive medications such as sodium nitroprusside or nitroglycerine to rapidly lower BP and mitigate end-organ damage.

The initial surgical procedure for phaeochromocytoma involves an open laparotomy and total adrenal gland excision. This method is now the standard of care due to its many advantages, including reduced operation time, complication rates, and hospital stays [172]. Individuals with synchronous bilateral phaeochromocytomas who are having simultaneous bilateral adrenalectomy can benefit from a cortical-sparing strategy for patients with mainly benign disease, as in *VHL* or *MEN-2*, but is contraindicated where there is a metastatic tendency as in *SDHx*-mutated patients. Additional treatment methods, such as stereotactic radiosurgery and external radiation therapy, are often necessary when managing head-and-neck paragangliomas [186].

Metastatic PPGLs cannot currently be cured, but they may be indolent and survival for many years is not infrequent. Therefore, the treatment aims to alleviate symptoms caused by an excess of catecholamines, local mass effect, pain from metastases, and the overall burden of the tumour [187, 188]. This is especially the case for tumours seen in young people, with *SDH-B* mutated tumours most likely be metastatic. In such cases treatment may be delayed until there is clear progression, with increasingly early use of somatostatin analogues or peptide receptor radionuclide therapy (PRRT) with ^177^Lu-dotatate, which has increasingly replaced ^131^I-mIBG therapy. For more rapidly progressive disease or where the tumour is unsuitable for radionuclide therapy, temozolomide or CVD (cyclophosphamide/vincristine/dacarbazine) can be used.

The long-term outcomes and quality of life for children with pheochromocytoma and catecholamine-secreting paraganglioma following surgical resection and medical therapy are influenced by a number of factors, including tumour characteristics, genetic predispositions and the necessity for ongoing monitoring. Surgical resection remains the primary treatment option, with long-term follow-up essential due to the risk of recurrence. It is recommended that children with a germline pathogenic or probable pathogenic variant in a PPGL predisposition gene be offered lifelong clinical follow-up [174].

## Peripheral glucocorticoid and mineralcorticoid pathway disorders

### Apparent mineralcorticoid excess

#### Epidemiology

Apparent mineralocorticoid excess (AME) is an extremely rare monogenic juvenile hypertensive syndrome with a prevalence that remains uncertain but estimated as < 1/1,000,000, although it probably varies between populations, depending on the level of kinship and endogamy [189]Data from: Orphanet Reports. www.orpha.net). Approximately one hundred AME cases have been described clinically and genetically worldwide and reported in the literature so far [189](Data from: Orphanet Reports. www.orpha.net) [190]. No sex predominance is reported for the disease [189].

#### Pathogenesis

Cortisol and aldosterone are secreted from the zona fasciculata and glomerulosa of the adrenal cortex, respectively. Cortisol is secreted in larger physiologic amounts than aldosterone, being in orders of magnitude 1000 to 2000 times higher [191, 192]. Both hormones display the same binding affinity for the MR, potentially modulating blood pressure. Endogenous cortisol availability in aldosterone target tissues, such as the distal nephron, the colon epithelial cells, and the salivary glands, is modulated by the action of the enzyme 11β-dehydrogenase type 2, whose gene, *HSD11B2*, is located on chromosome 16q22.1 [191, 193–195]. Indeed, 11β-HSD2 catalyses the conversion of cortisol to the less active metabolite cortisone, thus protecting the MR from illicit cortisol activation [191, 193–195]. Thus, compromised 11β-HSD2 activity leads to over-activation of the MR by endogenous cortisol with consequent induction of renal sodium retention and a salt-sensitive increase in BP. There are several causes of compromised 11β-HSD2 activity, including gene mutations or inhibition of this enzyme by xenobiotics such as the glycyrrhizic acid, an ingredient of liquorice.

**Fig. 1:**
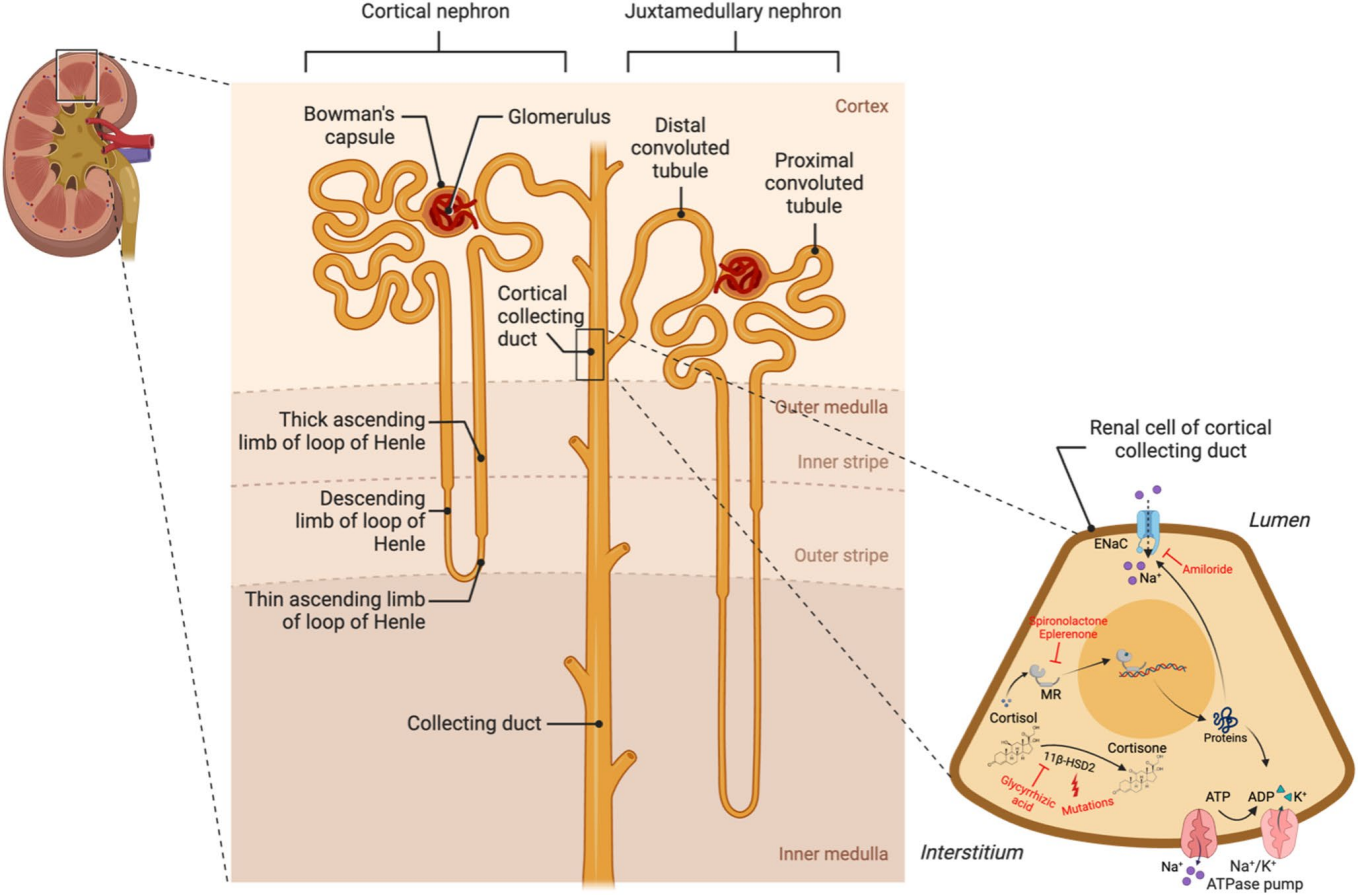
Cell mechanisms and therapy in Apparent Mineralcorticoid Excess. In renal cells of the cortical collecting duct (CCD), when 11β-HSD2 activity is decreased, as a consequence of inherited or acquired mutations, intracellular cortisol level increases, leading to activation of the MR. The binding of the mineralocorticoid receptor (MR) to its hormone response element on DNA increases the transcription of genes encoding specific aldosterone-inducible proteins, such as the rate-limiting subunits of the apical epithelial sodium channel (ENaC) and basolateral Na/K-ATPase, thus producing Na^+^ retention, volume expansion and hypertension. Spironolactone and eplerenone are MR antagonists. Amiloride is an ENaC blocker. Created with Biorender.com.

AME is an inherited autosomal recessive disorder caused by homozygous or compound heterozygous loss-of-function mutations or deletions in the *HSD11B2* gene, which has been mapped to chromosome 16q22 and consists of five exons (Data from: Orphanet Reports. www.orpha.net.) [190, 196]. Most of the known 51 mutations are found in exons 3, 4 or 5 of the *HSD11B2* gene, rarely in exon 1 and exon 2 [197]. A few mutations were found to not alter the amino-acid sequence but were potentially implicated in aberrant splicing [198]. In all cases, these mutations lead to abolition or a marked decrease in the 11β-HSD2 activity. Furthermore, the presence of single nucleotide polymorphisms, variations in microsatellite regions and epigenetic modifications in the *HSD11B2* gene, can affect its expression and thus prejudice the 11βHSD2 enzyme activity (Fig. 1) [192, 199, 200].

#### Diagnosis

Mutations in the *HSD11B2* gene cause the AME [191, 193–195]. AME represents a rare form of pseudo-hyperaldosteronism characterised by very early-onset and severe hypertension, associated with low renin levels and hypoaldosteronism. Besides the clinical characteristics, the diagnosis is based on biochemical findings of hypokalaemia, metabolic alkalosis, hyporeninaemia, hypoaldosteronaemia, and the detection of a marked increase (10 to 100-fold) in the ratio of cortisol/cortisone and tetrahydrocortisol/tetrahydrocortisone in plasma and urine. Genetic sequencing or *HSD11B2* gene mutations is available to confirm the milder form of AME (non-classical AME) characterizsed by less marked hypertension and only mild abnormalities of cortisol metabolism (Data from: Orphanet Reports. www.orpha.net.) [194].

#### Clinical presentation

Generally, in AME, compromised 11βHSD2 enzyme activity results in overstimulation of the MR by cortisol, causing sodium retention, hypokalaemia, and salt-dependent hypertension. However, patients with *HSD11B2* mutations showed varying degrees of severity in terms of clinical and biochemical features, according to the residual in vivo activity of the mutant 11βHSD2 enzyme. Mutations can cause a spectrum of manifestations and severities of the disease and depending on AME phenotypic severity, two forms can be recognised: classic and non-classic AME. and non-classic AME.

Classical AME starts very early in childhood, and it is characterised by a severe phenotype with low birth weight, failure to thrive, poor growth, severe and refractory hypertension, hypokalaemia, suppressed plasma renin activity with hypoaldosteronemia, polyuria, polydipsia, metabolic alkalosis and nephrocalcinosis. Non-classical AME is commonly diagnosed in adolescents or adults who develop the disease much later. than children with classic AME. Patients with non-classical AME present milder phenotypes including slight hypertension, increased biomarkers of renal and endothelial damage, like microalbuminuria and plasminogen activator inhibitor-1, and increased inflammation parameters, such as C-reactive protein and TNF-α [196–198].

#### Treatment

Treatment of AME consists of the use of MRAs and ENaC blockers such as spironolactone or eplerenone and amiloride, respectively, along with dietary sodium restriction and potassium supplementation to ameliorate hypertension (Fig. 1) [191, 194].

### Liddle syndrome

In the distal nephron, the two main Na-transporting cell types are the Distal Convoluted Tubule (DCT) and the Principal Cells (PS). In the first group of cells, the luminal Na-transporter is the thiazide sensitive sodium chloride (NaCl) cotransporter (NCC) [194, 201]. The electroneutral reabsorption of sodium, particularly in the early distal tubule, is predominantly facilitated by the phosphorylation of the thiazide-sensitive sodium chloride cotransporter (NCC) through a complex network of kinases, with regulation by angiotensin II and aldosterone through mechanisms that are still not fully understood [202].

In the Principal cell, sodium transportation is mediated by the ENaC [194], which is composed of three subunits, named α, β and γ. ENaC participates in a process that involves transepithelial ionic transport between the lumen of the renal collecting duct and the blood. Through its opening, Na + is actively transported across the basolateral membrane via the Na+/K+-ATPase, which operates in exchange for potassium ions [203]. At the physiological level, the channel is positively regulated by aldosterone and antidiuretic hormone [204].

Among monogenic forms of hypertension, mutations involving distal nephron Na + transport, such as Liddle Syndrome and Gordon Syndrome, play a significant role.

#### Epidemiology

Liddle syndrome (LS) is a juvenile form of genetic hypertension caused by point mutations of the *ENaC*, that cause aldosterone-independent sodium reabsorption in the distal nephron [205]. LS is a rare condition, and data describing formally diagnosed patients reported only 30 cases until 2008 [206]. Nevertheless, it may be considered the most common type of monogenic hypertension, being reported in many ethnic populations, including Caucasians, Asians, and Black people [194]. The prevalence across the general population of hypertensive young population is uncertainly reported at between 1.52% and 0.89% in two recent Chinese studies [207, 208].

#### Pathogenesis and Genetics

LS consists of a genetically determined defect in the distal tubular transport system, in which gain-of-function mutations of *ENaC* cause a constitutive activation of distal tubular sodium and water absorption [201]. This results in volume expansion and hypertension, onsetting at a young age. The disease is inherited in an Mendelian autosomal dominant fashion [209]. The causative point mutations are highly diverse, mostly involving the *ENaC β* (or *SCNN1B*) and *γ* (*SCNN1G*) subunits genes, located in 16p12 chromosome [210], although a mutation in *SCNN1A* encoding for the α-subunit was recently described [211]. To date, so far, the discovered mutations are reported as missense, nonsense and frameshift mutations, before or within the interested codon [210].

Depending on the affected ENaC subunit, LS is classified into three types: type 1 (β subunit, OMIM #177200), type 2 (γ subunit, OMIM #618114), and type 3 (α subunit, OMIM #618126) [201]. The pathogenetic mechanism consists of deletions or substitutions in a short proline-rich amino acid sequence of the C-terminus of *SCNN1B* and *SCNN1G*, which leads to inability of β and γ subunits to bind to an intracellular ubiquitin protein ligase (Nedd4) that physiologically removes the channel from the cell surface. Thus, the increased exposition of ENaC channels on the cell surface enhances sodium reabsorption, aldosterone-independently [210].

#### Diagnosis

Diagnosis relies on clinical suspicion, triggered by findings of normo/hypertension and hypokalaemia in a juvenile age. Low renin and aldosterone levels typically result from volume expansion due to increased sodium reabsorption [205, 212]. A negative family history should not be an exclusion criterion, as *de novo* mutations have also been identified. A good response to ENaC Blockers can be considered as diagnostic confirmation. Genetic sequencing can confirm the diagnosis [206].

#### Presenting features

Clinical features associated with LS can be recognised in an early-onset resistant arterial hypertension, associated with hypokalaemia and kaliuresis, metabolic alkalosis, suppressed plasma renin activity and low serum aldosterone levels [194, 201]. The clinical portrait of uncontrolled hypertension could also be associated with cardiovascular complications, such as premature stroke, myocardial infarction, sudden death in young age [191, 213]. LS types 1 and 2 usually present in late childhood or adolescence, but they can also occur in infancy. Indeed, patients can be diagnosed very early, thus any infant presenting with hypertension and metabolic alkalosis, with or without hypokalaemia, should raise the suspicion for LS [214].

Apart from the classical presentation, cases can also be asymptomatic, being diagnosed late in adulthood [194, 201, 205]. Particularly, since approximately 7–8% of patients diagnosed with LS are normotensive [205], and considering the incomplete penetrance of the disease, it is important to screen for this condition in individuals presenting with refractory hypertension or isolated hypokalaemia, particularly when suppressed aldosterone and renin levels are associated with a strong family history of severe or refractory hypertension and/or hypokalaemia [215]. Notably, genotype-phenotype correspondence is not always maintained, even within extremely severe phenotypes and mild forms within members of the same family, probably due to unknown environmental influences.

#### Treatment

Amiloride is an ENaC blocker and the use of this drug is highly effective in treating LS, as well as triamterene, at a dosage of 2.5–40 mg daily for amiloride and 50–100 mg daily for triamterene. Conversely, MRAs such as spironolactone or eplerenone are ineffective, since ENaC constitutive activation is aldosterone-independent, the lack of response is also highly informative in the diagnostic context [212]. Dietary sodium intake restriction (typically less than 2 g NaCl daily) could improve the efficacy of ENaC blockers, and should be initiated as an additional therapy in all patients with LS. Along with hypokalaemia and hypertension, cardiac, neurological, renal, and ophthalmic sequelae of hypertension are common. Therefore, they should be assessed and treated when necessary [205].

### Gordon syndrome (or Pseudo-hypoaldosteronism type II or Familial hyperkalaemic Hypertension)

Gordon Syndrome (GS), a rare form of monogenic familial hypertension, typically associated with hyperkalaemia, which sets it apart from other syndromic forms of hypertension, which lead to hypokalaemia.

#### Epidemiology

GS is defined as a rare form of hypertension. Its true prevalence is unknown.

#### Pathogenesis and Genetics

Four genes associated with the regulation of the NaCl cotransporter NCC (thiazide-sensitive sodium chloride cotransporter in distal convoluted tubule cells) have been associated with Gordon phenotypes: *WNK1* and *WNK4*, which encode a family of WNK (with-no-lysin) kinases, and *KLHL3* and *CUL3*, encoding Kelch-like 3 protein and Cullin 3, respectively [216]. No activating mutations in the NCC itself have been found so far. Inheritance occurs mostly in an autosomal dominant fashion [217].

Five subtypes of PHAII have been described, designated A to E. Type IIA has been associated with chromosome region 1q31-q42 with no gene yet identified, PHAII-B with specific variations in the *WNK4* gene (OMIM # 601844) (17q21), and PHAII-C by mutations in the *WNK1* gene (OMIM # 605232) (12p12.3.). Moreover, germline mutations in *KLHL3* (OMIM # 605775) (5q31.2) and *CUL3* (OMIM # 603136) (2q36) are related to PHAII-D and E, respectively [218]. De-novo mutations have been also described.

Mutations in all these genes, with various mechanisms, constitutively activate NCC, resulting in abnormal increased salt reabsorption in distal tubule and salt-sensitive hypertension. Furthermore, hypertension in GS is not solely linked to an increase in sodium and fluid reabsorption. Indeed, vascular endothelial dysfunction should be taken into account, due to an increased vascular smooth muscle tone, mediated by CUL3 deletion [219].

#### Presenting features

Diagnostic features, in addition to hypertension (not always present), are severe hyperkalaemia (reaching 8–9 mmol/L), which can lead to periodic paralysis, and metabolic hyperchloraemic acidosis without renal failure. Biochemical dosages show plasma renin activity suppression and variable aldosterone levels. The severity of hypertension, hyperkalaemia and metabolic acidosis varies according to different mutations [220], with an evident phenotype-genotype correlation, and some families or sporadic cases presented with milder symptoms [191]. Hypertension generally occurs in adolescence to adulthood. Rarely, neonatal presentations have been reported [221]. Notably, Spitzer-Weinstein syndrome in children is currently considered an early manifestation of GS in infancy, due to their similar clinical features [194].

Particularly in WNK4 mutations, hypercalciuria is also frequently found, even prior to development of hypertension [222], along with a decreased bone mineral density and without elevated parathyroid hormone or serum calcium [206, 216]. Furthermore, patients might show short stature and abnormal growth rate [223], dental abnormalities and intellectual disability [224].

#### Diagnosis

While PHA types 2 A, 2B, and 2 C are usually diagnosed in adolescence and adulthood, defects in *CUL3* (type 2E) and autosomal recessive *KLHL*,*3* mutations can present in infancy [225]. There are no formal diagnostic criteria for PHA II, with genetic investigations confirming the diagnosis.

#### Treatment

Hypertension in GS is strongly sensitive to salt restriction (20 mmol/d) [191], which can reverse symptoms [212]. Notably, since thiazide diuretics have a direct inhibitory effect on NCC, with low doses (50% of the age- and weight-adjusted dosage) being sufficient to achieve an excellent clinical and biochemical response, even for a long-term treatment [226].

## Familial or sporadic primary generalised glucocorticoid resistance or Chrousos syndrome

### Allostasis through a defective glucocorticoid receptor

At the cellular level, the actions of glucocorticoids are mediated by the glucocorticoid receptor (GR). The human GR (hGR) belongs to the steroid/thyroid/retinoic acid superfamily of nuclear receptors, and functions as a ligand-dependent transcription factor that regulates the expression of glucocorticoid responsive genes positively or negatively. The hGR is expressed by the *NR3C1* gene, which is located on chromosome 5 (5q31.3), and contains 10 exons [227, 228]. Within the target cell, glucocorticoids signal through their cognate receptor, which is located mainly in the cytoplasm, and forms a protein complex with immunophillins and heat shock proteins. The structure of the receptor changes, allowing the GR to move to the nucleus. In the nucleus, the ligand-activated hGRα forms dimers (homo-or heterodimers) that bind to specific DNA sequences, which are known as glucocorticoid response elements (GREs). GREs are located in the regulatory or promoter regions of glucocorticoid-responsive genes, therefore modulating their expression.

In humans, the glucocorticoid signal transduction pathway exerts its functions in inverted U-shaped activity-effect curve. The optimal effect contributes to homeostasis or eustasis and is achieved in the middle of glucocorticoid signalling activity. Suboptimal effects may be present on each side of the inverted U-shaped curve and can cause insufficient adaptation. This state is termed allostasis or cacostasis [228]. These conditions may present with clinical manifestations of glucocorticoid resistance. Mutations of the *NR3C1* gene can cause a rare familial or sporadic condition known as Chrousos syndrome, characterised by generalised or partial, target-tissue insensitivity to glucocorticoids, which leads to compensatory activation of the HPA axis and hypersecretion of ACTH in the systemic circulation. In Chrousos syndrome the clinical manifestations range from asymptomatic cases to severe cases of mineralocorticoid and/or androgen excess. In 2018, Vitellius et al. provided an estimation of the prevalence of *NR3C1* mutations in 100 patients with bilateral adrenal hyperplasia, increased arterial pressure, and/or hypercortisolism, but without any stigmata of Cushing’s syndrome. They found that five of these patients (5%) carried novel heterozygous *NR3C1* mutations [229].

#### Pathogenesis

The molecular basis of Chrousos syndrome has been ascribed primarily to mutations in the hGR gene, encoded by the *NR3C1* gene, which is located on the short arm of chromosome 5, and contains 10 exons. Many different mutations were identified, and in vitro studies showed that each mutation can present with: impaired ability to bind to DNA, more time required to translocate into the nucleus following ligand-induced activation, lower affinity for the ligand than the wild-type receptor, decreased ability to transactivate glucocorticoid-responsive genes, and a marked delay to translocate into the nucleus and interacted with the GRIP1 coactivator mostly through its AF-1 domain. This results in impairment of the molecular mechanisms of hGR action and decreased tissue sensitivity to glucocorticoids [228, 230].

#### Clinical presentation

The decreased tissue responsiveness to glucocorticoids leads to compensatory activation of the HPA axis causing hypersecretion of ACTH. Adrenal cortex hypertrophy results and activates the biosynthetic pathway of cortisol, adrenal androgens [androstenedione, dehydroepiandrosterone (DHEA), and DHEA-sulfate (DHEAS)], and steroid precursors with mineralocorticoid activity (deoxycorticosterone and corticosterone).

Therefore, patients may be asymptomatic or may present with clinical manifestations of mineralocorticoid excess (hypertension and/or hypokalaemic alkalosis) and/or androgen excess (ambiguous genitalia in karyotypic females, precocious puberty, acne, hirsutism, male-pattern hair loss, and hypofertility in both sexes, oligo-amenorrhoea and menstrual irregularities in women, and oligospermia in men).

#### Diagnosis

In addition to documenting various clinical manifestations, gathering personal and family medical history is crucial. The endocrine assessment involves measuring serum cortisol levels at 08:00 AM, plasma ACTH, plasma renin activity (while recumbent), serum aldosterone, androgens (testosterone, androstenedione, DHEA, DHEAS), and insulin. The biochemical evaluation includes determining levels of total cholesterol, HDL, LDL, triglycerides, and fasting glucose. The 24-hour urinary free cortisol (UFC) excretion should be measured over 2 or 3 consecutive days. Serum cortisol levels can be up to seven times higher than the upper limit of the normal range, while 24-hour UFC excretion can be up to fifty times higher than the upper normal range. ACTH levels may be normal or elevated.

The dexamethasone suppression test involves administering increasing doses of dexamethasone (0.3, 0.6, 1.0, 1.5, 2.0, 2.5, 3.0 mg) orally at midnight on alternate days, with serum cortisol and dexamethasone levels measured at 08:00 AM the following morning. It is also important to consider measuring serum dexamethasone levels concurrently to rule out non-adherence to treatment, increased metabolic clearance, or reduced absorption of the medication.

Two primary molecular biology methods confirm the diagnosis of Primary Generalised Glucocorticoid Resistance: dexamethasone-binding assays and thymidine incorporation assays [228, 231].

#### Therapy

The main objective in Chrousos syndrome is to suppress ACTH, thus lowering the increased production of adrenal steroids with mineralocorticoid and androgenic activity. Treatment consists of the administration of high doses of dexamethasone (1–3 mg given once daily at night), which suppress the endogenous secretion of ACTH in affected subjects. Dexamethasone should be carefully titrated according to the clinical manifestations and biochemical profile of the patients. It is crucial to achieve adequate suppression of the HPA axis to prevent the development of an ACTH-secreting adenoma [231].

## Conclusions

The diagnosis and management of endocrine hypertension during childhood and transition age still represent a significant clinical challenge today [232]. Biochemical and clinical aspects to be considered in the differential diagnosis of adrenal causes of hypertension, and brief hints about treatment are summarised in Table 1. [23]. Arterial hypertension can lead to cardiovascular morbidity, mortality, and organ damage, and when it occurs during childhood or the transition to adulthood, secondary causes of hypertension have to be investigated, diagnosed early and treated.

**Table 1:** Summary table adrenal causes of hypertension during childhood and adolescence

Adrenal Disorder	Germinal mutations gene	Age at hypertension onset	Gender prevalence	K^+^	Renin	Aldosteron	Cortisol	ACTH	Other suggestive biochemical aspects	Suggestive clinical aspect	Treatment	References
11β-hydroxylase deficiency	*CYP11B1*	Variable: 0–17 yrs	–	↓	↓	↓	N/↓	↑	↑ DOC, Increased 17 OH PRG, 11- deoxycortisol, androstenedione, testosterone, DHEA-S	Ambiguous genitalia, Virilization/ PCOS phenotype in F, Precocious puberty, short stature, TART in M	MRAs, ENaCi Cortisol substitution if needed Antiandrogens if needed K^+^implementation if needed	15–34, 235
17-hydroxylase/17,20-lyase deficiency	*CYP17A1*	Variable: Mainly adolescence	–	↓	↓	N/↑	↓	↑	↑ Corticosterone ↑ DOC ↑ Pregnenolone ↑ Progesterone ↓/N androstenedione ↓/N testosterone ↓/N DHEA-S ↓/N 17OHprogesterone	Abnormal sexual differentiation in M Lack of breast development, Ovarian cysts, Amenorrhea and Infertility in F	MRAs Te in M EP in F	35–51
FH1	*CYP11B1-CYP11B2* *Chimeric gene*	Variable: < 20 yrs	–	N/↓	↓	↑	N	N	↑ 18-oxo-cortisol	Micro/macro vascular complications, cardiac remodeling, Myocardial infarction and stroke at young age	Low dose GCs MRAs K^+^implementation if needed	55–67, 235
FH2	*CLCN2*	Variable: < 20 yrs	–	N/↓	↓	↑	N	N	Alkalosis		MRAs K^+^implementation if needed, adrenalectomy if needed	56–57, 68–75,
FH3	*KCNJ5*	Variable: 5yrs-adult age	–	N/↓	↓	↑	N	N	Alkalosis	**Severe phenotype**: Early-onset drug resistant hypertension, Polyuria, Polydipsia, Myalgia, Growth retardation **Milder phenoptype**: milder/later-onset hypertension showing favorable disease progression during growth	MRAs and other antihypertensive drugs K^+^implementation if needed, Adenalectomy if needed	56–57, 69, 76–92
FH4	*CACNA1H*	Variable: < 10 yrs-adult age	–	N/↓	↓	↑	N	N	Alkalosis	**Severe phenotype**: Mental retardation, growth retardation, learning disorders **Milder phenotype**: no PA/PA in adult age	MRAs K^+^implementation if needed	56–57, 93–99
CANCA1D- related hyperaldosteronism	*CACNA1D*	Variable: 0-5yrs	–	↓	↓	↑	N	N	Hyperinsulinemia hypoglycemia Alkalosis	Cardiac abnormalities, Congenital deafness, Neonatal hypoglycemia	Dihydropyridine Ca^++^ antagonists	57, 100–108
CS	*PRKAR1*,*GNAS*,* MEN1*,* TP53*,* MSH2*,* MLH1*,* PMS2*,* MSH6*,* EPCAM*	Variable: (if < 5 yrs consider mainly CS)	F > M	N/↓	N	N	↑	↓ in CS N/↑ in CD	↑24 h UFC ↑ LNSC ↑midnight serum cortisol ↑morning serum cortisol after DST	Cushingoid phenotype: facial rounding, weight gain, growth retardation and increased virilization	Steroidogenesis inhibitors (Ketoconazole, Osilodrostat) Low dose mitotane (Caution in use of medical treatments: surgery to be preferred when feasible)	109–130, 141–155, 235
PPGL	*SDHx*,* VHL*,* SDH*,* MEN2*,* NF1*	Variable	–	N	N	N	N	N	↑ Plasma metanephrines ↑ Urine metanephrines	Severe headache, Palpitations, Sweating, Pallor, Abdominal pain, Tremors, Weight loss	**Perioperative treatment**: Phenoxybenzamine or Phentolamine, ꞵ-blockers if needed **Hypertensive crisis**: Sodium nitroprusside, Nitroglycerin or other antyhypertensive drugs	164–188
Apparent mineralcorticoid excess	*HSD11B2*	Variable: Very early childhood/adolescent/ adult age	No	N/↓	↓	↑	N	N	Alkalosis	**Severe phenotype**: Low birth weight, Failure to thrive, Poor growth, Alkalosis, Nephrocalcinosis, Polyuria, Polydipsia **Mild phenotype**: Slight/late onset hypertension	MRAs/ENaCi K^+^implementation if needed	192–194, 196, 199

Among the potential causes of hypertension at these ages, adrenal disorders such as enzyme defects in steroidogenesis, FH and CS, lead to an accumulation of hormones or steroidogenesis metabolites with mineralocorticoid activity, resulting in various degrees of severity of hypertension associated with hypokalaemia and sometimes alkalosis. The forms of congenital adrenal hyperplasia causing hypertension may mask inadequate glucocorticoid secretion, leading to the risk of adrenal crises under stress conditions, and therefore deserve attention for the evaluation of possible glucocorticoid replacement therapy.

If the steroidogenetic pathway leading to the production of androgens is enhanced, a clinical picture of congenital genital ambiguity with virilisation in females and hyperandrogenism can occur. On the contrary, when the deficient enzymatic step is necessary for the production of sexual steroids, genital ambiguity with feminisation in the male and amenorrhea and a PCOS-like syndrome in the female can occur.

A picture of hypertension accompanied by genital ambiguity at birth or alterations of puberty in males or amenorrhoea in females must therefore raise the suspicion of CAH. The various forms of familial hyperaldosteronism can present with a notable variability in the severity of hypertension, of the associated hypokalemia and cardiovascular damage, with incomplete penetrance or various patterns of mosaicism contributing to the heterogeneity of the clinical and biochemical characteristics.

The presence of peculiar clinical characteristics such as hyperinsulinaemic hypoglycaemia at birth or neuropsychiatric manifestations such as those of PASNA or congenital cardiac anomalies can direct investigations towards the diagnostic suspicion of a specific type of FH. The response to medical treatment can also influence the type of FH (good clinical response to GCS or CA antagonists). Definitive diagnosis requires genetic tests aimed at searching for the above-mentioned germline mutations.

The early diagnosis and the extension of molecular investigations to family members, even if asymptomatic, are necessary as the onset of FH can be late and FH can expose the risk of cardiovascular events at a young age. The presence of a Cushingoid phenotype, often associated with excessive obesity but poor growth, especially in association with a dysmetabolic pattern, must raise clinical suspicion of endogenous hypercortisolism, and early diagnosis and treatment are essential, given the long-term comorbidities associated with chronic hypercortisolism. Morphological investigation should be performed in all cases of hypercortisolism to exclude the rare malignancies and to identify specific morphological adrenal characteristics such as pigmented nodular adrenocortical disease.

Phaeochromocytomas and paragangliomas, quite frequent in children with hypertension, present with a peculiar clinical picture compared to other forms of adrenal hypertensions and require a multidisciplinary approach in evaluating the appropriate treatment and in-depth biochemical and molecular investigations to exclude a systemic genetic syndrome. Prompt identification of adrenal causes of secondary hypertension is crucial for timely and effective management, addressing systemic manifestations such as electrolyte imbalances and early cardiovascular events associated with hypertension. In conclusion, this review underlines the importance of recognising the clinical characteristics suggestive of adrenal pathologies causing endocrine hypertension and of promptly directing these patients to referral centres for the advanced diagnosis and the personalised treatment of these rare pathologies.

## References

[1] Nguyen TN, Chow CK (2021) Global and National high blood pressure burden and control. Lancet Lond Engl 398:932–933 10.1016/S0140-6736(21)01688-3 34450082

[2] Mancia G, Kreutz R, Brunström M, Burnier M, Grassi G, Januszewicz A et al (2023) 2023 ESH guidelines for the management of arterial hypertension the task force for the management of arterial hypertension of the European society of hypertension: endorsed by the international society of hypertension (ISH) and the European renal association (ERA). J Hypertens 41:1874–2071 37345492 10.1097/HJH.0000000000003480

[3] Lurbe E, Agabiti-Rosei E, Cruickshank JK, Dominiczak A, Erdine S, Hirth A, Invitti C, Litwin M, Mancia G, Pall D, Rascher W (2016) 2016 European Society of Hypertension guidelines for the management of high blood pressure in children and adolescents. Journal of hypertension 34(10):1887–1920 27467768 10.1097/HJH.0000000000001039

[4] Stergiou GS, Palatini P, Parati G, O’Brien E, Januszewicz A, Lurbe E et al (2021) 2021 European Society of Hypertension practice guidelines for office and out-of-office blood pressure measurement. J Hypertens [Internet]. [cited 2024 Oct 9];39:1293–302. Available from: https://journals.lww.com/10.1097/HJH.0000000000002843 10.1097/HJH.0000000000002843 33710173

[5] Rao G, Diagnosis Epidemiology, and Management of Hypertension in Children. Pediatrics [Internet]. 2016 [cited 2024 Oct 9];138:e20153616. Available from: https://publications.aap.org/pediatrics/article/138/2/e20153616/52413/Diagnosis-Epidemiology-and-Management-of 10.1542/peds.2015-3616 27405770

[6] Dormanesh B, Arasteh P, Daryanavard R, Mardani M, Ahmadi M, Nikoupour H (2023) Epidemiology of obesity and high blood pressure among school-age children from military families: the largest report from our region. BMC Pediatr [Internet]. [cited 2024 Oct 9];23:37. Available from: https://bmcpediatr.biomedcentral.com/articles/10.1186/s12887-023-03839-z 10.1186/s12887-023-03839-z PMC9868491 36683049

[7] Maldonado J, Pereira T, Fernandes R, Santos R, Carvalho M (2011) An approach of hypertension prevalence in a sample of 5381 Portuguese children and adolescents. The AVELEIRA registry. Hypertension in Children. Blood Press [Internet]. [cited 2024 Oct 9];20:153–7. Available from: http://www.tandfonline.com/doi/full/10.3109/08037051.2010.542649 10.3109/08037051.2010.542649 21142582

[8] Chiolero A, Cachat F, Burnier M, Paccaud F, Bovet P (2007) Prevalence of hypertension in schoolchildren based on repeated measurements and association with overweight. J Hypertens [Internet]. [cited 2024 Oct 9];25:2209–17. Available from: https://journals.lww.com/00004872-200711000-00007 10.1097/HJH.0b013e3282ef48b2 17921814

[9] Viggiano D, De Filippo G, Rendina D, Fasolino A, D’Alessio N, Avellino N et al Screening of Metabolic Syndrome in Obese Children: A Primary Care Concern. J Pediatr Gastroenterol Nutr [Internet]. 2009 [cited 2024 Oct 9];49:329–34. Available from: https://onlinelibrary.wiley.com/doi/10.1097/MPG.0b013e31819b54b7 10.1097/MPG.0b013e31819b54b7 19590449

[10] Jacobs DR, Woo JG, Sinaiko AR, Daniels SR, Ikonen J, Juonala M et al (2022) Childhood Cardiovascular Risk Factors and Adult Cardiovascular Events. N Engl J Med [Internet]. [cited 2024 Oct 9];386:1877–88. Available from: http://www.nejm.org/doi/10.1056/NEJMoa2109191 10.1056/NEJMoa2109191 PMC9563825 35373933

[11] De Silva T, Cosentino G, Ganji S, Riera-Gonzalez A, Hsia DS Endocrine Causes of Hypertension. Curr Hypertens Rep [Internet]. 2020 [cited 2024 Oct 9];22:97. Available from: https://link.springer.com/10.1007/s11906-020-01108-3 10.1007/s11906-020-01108-3 33079272

[12] Zennaro M-C, Boulkroun S, Fernandes-Rosa FL (2020) Pathogenesis and treatment of primary aldosteronism. Nat Rev Endocrinol [Internet]. [cited 2024 Oct 9];16:578–89. Available from: https://www.nature.com/articles/s41574-020-0382-4 10.1038/s41574-020-0382-4 32724183

[13] Ivy JR, Oosthuyzen W, Peltz TS, Howarth AR, Hunter RW, Dhaun N et al Glucocorticoids Induce Nondipping Blood Pressure by Activating the Thiazide-Sensitive Cotransporter. Hypertension [Internet]. 2016 [cited 2024 Oct 9];67:1029–37. Available from: https://www.ahajournals.org/doi/10.1161/HYPERTENSIONAHA.115.06977 10.1161/HYPERTENSIONAHA.115.06977 PMC4905621 26953322

[14] Fernandez CJ, Hanna FWF, Pacak K, Nazari MA (2023) Catecholamines and blood pressure regulation. Endocr Hypertens [Internet]. Elsevier; [cited 2024 Oct 9]. pp. 19–34. Available from: https://linkinghub.elsevier.com/retrieve/pii/B9780323961202000108

[15] Merke DP, Bornstein SR (2005) Congenital adrenal hyperplasia. The Lancet [Internet]. [cited 2024 Oct 9];365:2125–36. Available from: https://linkinghub.elsevier.com/retrieve/pii/S0140673605667360 10.1016/S0140-6736(05)66736-0 15964450

[16] BONGIOVANNI AM, EBERLEIN WR (1956) Plasma and urinary corticosteroids in the hypertensive form of congenital adrenal hyperplasia. J Biol Chem 223:85–94 13376579

[17] Li J, Zhang F, Xu M, Qiu H, Zhou C, Li L et al (2023) Case Report: A combination of chimeric CYP11B2/CYP11B1 and a novel p.Val68Gly CYP11B1 variant causing 11β-Hydroxylase deficiency in a Chinese patient. Front Endocrinol [Internet]. [cited 2024 Oct 9];14:1216767. Available from: https://www.frontiersin.org/articles/10.3389/fendo.2023.1216767/full 10.3389/fendo.2023.1216767 PMC10679387 38027139

[18] Matsubara K, Kataoka N, Ogita S, Sano S, Ogata T, Fukami M et al (2014) Uniparental disomy of chromosome 8 leading to homozygosity of a *CYP11B1* mutation in a patient with congenital adrenal hyperplasia: Implication for a rare etiology of an autosomal recessive disorder. Endocr J [Internet]. [cited 2024 Oct 9];61:629–33. Available from: https://www.jstage.jst.go.jp/article/endocrj/61/6/61_EJ13-0509/_article 10.1507/endocrj.ej13-0509 24621779

[19] Valadares LP, Pfeilsticker ACV, De Brito Sousa SM, Cardoso SC, De Moraes OL, Gonçalves, De Castro LC et al (2018) Insights on the phenotypic heterogenity of 11β-hydroxylase deficiency: clinical and genetic studies in two novel families. Endocrine [Internet]. [cited 2024 Oct 9];62:326–32. Available from: http://link.springer.com/10.1007/s12020-018-1691-4 10.1007/s12020-018-1691-4 30242600

[20] Nimkarn S, New MI (2008) Steroid 11β- hydroxylase deficiency congenital adrenal hyperplasia. Trends Endocrinol Metab [Internet]. [cited 2024 Oct 9];19:96–9. Available from: https://linkinghub.elsevier.com/retrieve/pii/S1043276008000088 10.1016/j.tem.2008.01.002 18294861

[21] White PC, STEROID 11β-HYDROXYLASE, DEFICIENCY AND RELATED DISORDERS (2001). Endocrinol Metab Clin North Am [Internet]. [cited 2024 Oct 9];30:61–79. Available from: https://linkinghub.elsevier.com/retrieve/pii/S0889852908700197 10.1016/s0889-8529(08)70019-7 11344939

[22] Mimouni M, Kaufman H, Roitman A, Morag Ch, Sadan N (1985) Hypertension in a neonate with 11?-hydroxylase deficiency. Eur J Pediatr [Internet]. [cited 2024 Oct 9];143:231–3. Available from: http://link.springer.com/10.1007/BF00442149 10.1007/BF00442149 3872797

[23] Zachmann M, Tassinari D, Prader A Clinical and Biochemical Variability of Congenital Adrenal Hyperplasia Due to llβ-Hydroxylase Deficiency, A Study of 25 Patients*. J Clin Endocrinol Metab [Internet]. 1983 [cited 2024 Oct 9];56:222–9. Available from: https://academic.oup.com/jcem/article-lookup/doi/10.1210/jcem-56-2-222 10.1210/jcem-56-2-222 6296182

[24] Chabre O, Portrat-Doyen S, Chaffanjon P, Vivier J, Liakos P, Labat-Moleur F et al Bilateral Laparoscopic Adrenalectomy for Congenital Adrenal Hyperplasia with Severe Hypertension, Resulting from Two Novel Mutations in Splice Donor Sites of CYP11B1. J Clin Endocrinol Metab [Internet]. 2000 [cited 2024 Oct 9];85:4060–8. Available from: https://academic.oup.com/jcem/article/85/11/4060/2852684 10.1210/jcem.85.11.6897 11095433

[25] Hague WM, Honour JW, MALIGNANT HYPERTENSION IN CONGENITAL ADRENAL HYPERPLASIA DUE TO 11β-HYDROXYLASE DEFICIENCY. Clin Endocrinol (Oxf) [Internet]. 1983 [cited 2024 Oct 9];18:505–10. Available from: https://onlinelibrary.wiley.com/doi/10.1111/j.1365-2265.1983.tb02880.x 10.1111/j.1365-2265.1983.tb02880.x 6603291

[26] John M, Menon SK, Shah NS, Menon PS (2009) Congenital adrenal hyperplasia 11beta-hydroxylase deficiency: two cases managed with bilateral adrenalectomy. Singap Med J 50:e68–70 19296015

[27] Rösler A, Leiberman E, Cohen T High frequency of congenital adrenal hyperplasia (classic 11β-hydroxylase deficiency) among Jews from Morocco. Am J Med Genet [Internet]. 1992 [cited 2024 Oct 9];42:827–34. Available from: https://onlinelibrary.wiley.com/doi/10.1002/ajmg.1320420617 10.1002/ajmg.1320420617 1554023

[28] Khattab A, Haider S, Kumar A, Dhawan S, Alam D, Romero R et al (2017) Clinical, genetic, and structural basis of congenital adrenal hyperplasia due to 11β-hydroxylase deficiency. Proc Natl Acad Sci [Internet]. [cited 2024 Oct 9];114. Available from: 10.1073/pnas.1621082114 10.1073/pnas.1621082114 PMC5347606 28228528

[29] Storr HL, Barwick TD, Snodgrass GAI, Booy R, Morel Y, Reznek RH et al (2003) Hyperplasia of Adrenal Rest Tissue Causing a Retroperitoneal Mass in a Child with 11β-Hydroxylase Deficiency. Horm Res Paediatr [Internet]. [cited 2024 Oct 9];60:99–102. Available from: https://karger.com/HRE/article/doi/10.1159/000071878 10.1159/000071878 12876421

[30] Kaynar M, Sönmez MG, Ünlü Y, Karatağ T, Tekinarslan E, Sümer A (2014) Testicular Adrenal Rest Tumor in 11-Beta-Hydroxylase Deficiency Driven Congenital Adrenal Hyperplasia. Korean J Urol [Internet]. [cited 2024 Oct 9];55:292. Available from: https://icurology.org/DOIx.php?id=10.4111/kju.2014.55.4.292 10.4111/kju.2014.55.4.292 PMC3988443 24741421

[31] Ahmad IC, Yilmaz TF, Kocakoç E (2014) Doppler ultrasonography and magnetic resonance imaging findings of testicular adrenal rest tissue in a patient with 11 β hydroxilase deficiency. Case report. Med Ultrason 16:383–385 25463895

[32] Aycan Z, Bas VN, Cetinkaya S, Yilmaz Agladioglu S, Tiryaki T (2013) Prevalence and long-term follow‐up outcomes of testicular adrenal rest tumours in children and adolescent males with congenital adrenal hyperplasia. Clin Endocrinol (Oxf) [Internet]. [cited 2024 Oct 9];78:667–72. Available from: https://onlinelibrary.wiley.com/doi/10.1111/cen.12033 10.1111/cen.12033 23057653

[33] Reisch N, Högler W, Parajes S, Rose IT, Dhir V, Götzinger J et al (2013) A Diagnosis Not to Be Missed: Nonclassic Steroid 11β-Hydroxylase Deficiency Presenting With Premature Adrenarche and Hirsutism. J Clin Endocrinol Metab [Internet]. [cited 2024 Oct 9];98:E1620–5. Available from: https://academic.oup.com/jcem/article-lookup/doi/10.1210/jc.2013-1306 10.1210/jc.2013-1306 23940125

[34] Sun B, Lu L, Gao Y, Yu B, Chen S, Tong A et al (2022) High prevalence of hypertension and target organ damage in patients with 11β-hydroxylase deficiency. Clin Endocrinol (Oxf) [Internet]. [cited 2024 Oct 9];96:657–65. Available from: https://onlinelibrary.wiley.com/doi/10.1111/cen.14677 10.1111/cen.14677 35067946

[35] Biglieri EG, Herron MA, Brust N (1966) 17-hydroxylation deficiency in man. J Clin Invest [Internet]. [cited 2024 Oct 9];45:1946–54. Available from: http://www.jci.org/articles/view/105499 10.1172/JCI105499 PMC292880 4288776

[36] Yanase T, Simpson ER, Waterman MR 17α-Hydroxylase/17,20-Lyase Deficiency: From Clinical Investigation to Molecular Definition*. Endocr Rev [Internet]. 1991 [cited 2024 Oct 9];12:91–108. Available from: https://academic.oup.com/edrv/article-lookup/doi/10.1210/edrv-12-1-91 10.1210/edrv-12-1-91 2026124

[37] Costa-Santos M, Kater CE, Auchus RJ, Two Prevalent CYP (2004) *17* Mutations and Genotype-Phenotype Correlations in 24 Brazilian Patients with 17-Hydroxylase Deficiency. J Clin Endocrinol Metab [Internet]. [cited 2024 Oct 9];89:49–60. Available from: https://academic.oup.com/jcem/article-lookup/doi/10.1210/jc.2003-031021 10.1210/jc.2003-031021 14715827

[38] Fontenele R, Costa-Santos M, Kater CE 17α-Hydroxylase Deficiency is an Underdiagnosed Disease: High Frequency of Misdiagnoses in a Large Cohort of Brazilian Patients. Endocr Pract [Internet]. 2018 [cited 2024 Oct 9];24:170–8. Available from: https://linkinghub.elsevier.com/retrieve/pii/S1530891X20354057 10.4158/EP171987.OR 29144824

[39] Miura K, Yasuda K, Yanase T, Yamakita N, Sasano H, Nawata H et al (1996) Mutation of cytochrome P-45017 alpha gene (CYP17) in a Japanese patient previously reported as having glucocorticoid-responsive hyperaldosteronism: with a review of Japanese patients with mutations of CYP17. J Clin Endocrinol Metab [Internet]. [cited 2024 Oct 9];81:3797–801. Available from: https://academic.oup.com/jcem/article-lookup/doi/10.1210/jcem.81.10.8855840 10.1210/jcem.81.10.8855840 8855840

[40] Zhang M, Sun S, Liu Y, Zhang H, Jiao Y, Wang W et al (2015) New, recurrent, and prevalent mutations: Clinical and molecular characterization of 26 Chinese patients with 17alpha-hydroxylase/17,20-lyase deficiency. J Steroid Biochem Mol Biol [Internet]. [cited 2024 Oct 9];150:11–6. Available from: https://linkinghub.elsevier.com/retrieve/pii/S096007601500045X 10.1016/j.jsbmb.2015.02.007 25697092

[41] Auchus RJ Steroid 17-hydroxylase and 17,20-lyase deficiencies, genetic and pharmacologic. J Steroid Biochem Mol Biol [Internet]. 2017 [cited 2024 Oct 9];165:71–8. Available from: https://linkinghub.elsevier.com/retrieve/pii/S0960076016300164 10.1016/j.jsbmb.2016.02.002 PMC4976049 26862015

[42] Zuber MX, Simpson ER, Waterman MR Expression of Bovine 17α-Hydroxylase Cytochrome P-450 cDNA in Nonsteroidogenic (COS 1) Cells. Science [Internet]. 1986 [cited 2024 Oct 9];234:1258–61. Available from: https://www.science.org/doi/10.1126/science.3535074 10.1126/science.3535074 3535074

[43] Flück CE, Tajima T, Pandey AV, Arlt W, Okuhara K, Verge CF et al (2004) Mutant P450 oxidoreductase causes disordered steroidogenesis with and without Antley-Bixler syndrome. Nat Genet [Internet]. [cited 2024 Oct 9];36:228–30. Available from: https://www.nature.com/articles/ng1300 10.1038/ng1300 14758361

[44] Miller WL (2004) P450 oxidoreductase deficiency: a new disorder of steroidogenesis with multiple clinical manifestations. Trends Endocrinol Metab [Internet]. [cited 2024 Oct 9];15:311–5. Available from: https://linkinghub.elsevier.com/retrieve/pii/S1043276004001535 10.1016/j.tem.2004.07.005 15350602

[45] Gregosiewicz A, Pietroń K, Stazka-Gregosiewicz E (1987) [Degeneration of the articular structures in patients with hemophilia. Etiology and pathomorphology of the changes and the radiological picture]. Chir Narzadow Ruchu Ortop Pol 52:442–446 3452497

[46] Kurnaz E, Kartal Baykan E, Türkyılmaz A, Yaralı O, Yavaş Abalı Z, Turan S et al (2020) Genotypic Sex and Severity of the Disease Determine the Time of Clinical Presentation in Steroid 17α-Hydroxylase/17,20-Lyase Deficiency. Horm Res Paediatr [Internet]. [cited 2024 Oct 9];93:558–66. Available from: https://karger.com/HRP/article/doi/10.1159/000515079 10.1159/000515079 33780934

[47] Pan P, Zheng L, Huang J, Chen X, Ni R, Zhang Q et al (2023) Endocrine profiles and cycle characteristics of infertile 17α-hydroxylase/17,20-lyase Deficiency Patients undergoing assisted Reproduction Treatment: a retrospective cohort study. J Ovarian Res [Internet]. [cited 2024 Oct 9];16:111. Available from: https://ovarianresearch.biomedcentral.com/articles/10.1186/s13048-023-01190-6 10.1186/s13048-023-01190-6 PMC10265862 37316894

[48] Xu Y, Jiang S, Yan Z, Niu Y, Du W, Liu B et al (2022) Phenotypic Heterogeneity and Fertility Potential of Patients With 17-Hydroxylase/17,20-lyase Deficiency. J Clin Endocrinol Metab [Internet]. [cited 2024 Oct 9];107:e2610–8. Available from: https://academic.oup.com/jcem/article/107/6/e2610/6511425 10.1210/clinem/dgac029 35043964

[49] Britten FL, Ulett KB, Duncan EL, Perry-Keene DA (2013) Primary amenorrhoea with hypertension: undiagnosed 17‐α‐hydroxylase deficiency. Med J Aust [Internet]. [cited 2024 Oct 9];199:556–8. Available from: https://onlinelibrary.wiley.com/doi/10.5694/mja12.11619 10.5694/mja12.11619 24138383

[50] Morimoto I, Maeda R, Izumi M, Ishimaru T, Nishimori I, Nagataki S An Autopsy Case of 17α-Hydroxylase Deficiency with Malignant Hypertension. J Clin Endocrinol Metab [Internet]. 1983 [cited 2024 Oct 9];56:915–9. Available from: https://academic.oup.com/jcem/article-lookup/doi/10.1210/jcem-56-5-915 10.1210/jcem-56-5-915 6300176

[51] Zhao Z, Lu L, Wang O, Wu X, Sun B, Zhang W et al (2022) High incidence of hypertension-mediated organ damage in a series of Chinese patients with 17α-hydroxylase deficiency. Endocrine [Internet]. [cited 2024 Oct 9];76:151–61. Available from: https://link.springer.com/10.1007/s12020-021-02966-w 10.1007/s12020-021-02966-w 35032013

[52] Araujo-Castro M, Martín Rojas-Marcos P, Parra Ramírez P Familial forms and molecular profile of primary hyperaldosteronism. Hipertens Riesgo Vasc [Internet]. 2022 [cited 2024 Oct 9];39:167–73. Available from: https://linkinghub.elsevier.com/retrieve/pii/S1889183722000538 10.1016/j.hipert.2022.05.007 35778363

[53] Mulatero P, Monticone S, Deinum J, Amar L, Prejbisz A, Zennaro M-C et al (2020) Genetics, prevalence, screening and confirmation of primary aldosteronism: a position statement and consensus of the Working Group on Endocrine Hypertension of The European Society of Hypertension ∗. J Hypertens [Internet]. [cited 2024 Oct 9];38:1919–28. Available from: https://journals.lww.com/10.1097/HJH.0000000000002510 10.1097/HJH.0000000000002510 32890264

[54] Scholl UI (2022) Genetics of Primary Aldosteronism. Hypertension [Internet]. [cited 2024 Oct 9];79:887–97. Available from: https://www.ahajournals.org/doi/10.1161/HYPERTENSIONAHA.121.16498 10.1161/HYPERTENSIONAHA.121.16498 PMC8997684 35139664

[55] Aglony M, Martínez-Aguayo A, Carvajal CA, Campino C, García H, Bancalari R et al Frequency of Familial Hyperaldosteronism Type 1 in a Hypertensive Pediatric Population: Clinical and Biochemical Presentation. Hypertension [Internet]. 2011 [cited 2024 Oct 9];57:1117–21. Available from: https://www.ahajournals.org/doi/10.1161/HYPERTENSIONAHA.110.168740 10.1161/HYPERTENSIONAHA.110.168740 21502562

[56] Lenzini L, Prisco S, Caroccia B, Rossi GP Saga of Familial Hyperaldosteronism: Yet a New Channel. Hypertension [Internet]. 2018 [cited 2024 Oct 9];71:1010–4. Available from: https://www.ahajournals.org/doi/10.1161/HYPERTENSIONAHA.118.11150 10.1161/HYPERTENSIONAHA.118.11150 29735637

[57] Staermose S, Marwick TH, Gordon RD, Cowley D, Dowling A, Stowasser M Elevated Serum Interleukin 6 Levels in Normotensive Individuals With Familial Hyperaldosteronism Type 1. Hypertension [Internet]. 2009 [cited 2024 Oct 9];53. Available from: https://www.ahajournals.org/doi/10.1161/HYPERTENSIONAHA.108.128512 10.1161/HYPERTENSIONAHA.108.128512 19221206

[58] Korah HE, Scholl UI (2015) An Update on Familial Hyperaldosteronism. Horm Metab Res [Internet]. [cited 2024 Oct 9];47:941–6. Available from: http://www.thieme-connect.de/DOI/DOI?10.1055/s-0035-1564166 10.1055/s-0035-1564166 26445452

[59] Stowasser M, Gartside MG, Taylor WL, Tunny TJ, Gordon RD (1997) In Familial Hyperaldosteronism Type I, Hybrid Gene-Induced Aldosterone Production Dominates That Induced by Wild-Type Genes ^1^. J Clin Endocrinol Metab [Internet]. [cited 2024 Oct 9];82:3670–6. Available from: https://academic.oup.com/jcem/article-lookup/doi/10.1210/jcem.82.11.4365 10.1210/jcem.82.11.4365 9360524

[60] Stowasser M, Bachmann AW, Huggard PR, Rossetti TR, Gordon RD Treatment of Familial Hyperaldosteronism Type I: Only Partial Suppression of Adrenocorticotropin Required to Correct Hypertension. J Clin Endocrinol Metab [Internet]. 2000 [cited 2024 Oct 9];85:3313–8. Available from: https://academic.oup.com/jcem/article/85/9/3313/2660633 10.1210/jcem.85.9.6834 10999827

[61] Stowasser M, Klemm SA, Tunny TJ, Gordon RD, PLASMA ALDOSTERONE RESPONSE, TO ACTH IN SUBTYPES OF PRIMARY ALDOSTERONISM. Clin Exp Pharmacol Physiol [Internet]. 1995 [cited 2024 Oct 9];22:460–2. Available from: https://onlinelibrary.wiley.com/doi/10.1111/j.1440-1681.1995.tb02044.x 10.1111/j.1440-1681.1995.tb02044.x 8582103

[62] Tan ST, Boyle V, Elston MS Systematic Review of Therapeutic Agents and Long-Term Outcomes of Familial Hyperaldosteronism Type 1. Hypertension [Internet]. 2023 [cited 2024 Oct 9];80:1517–25. Available from: https://www.ahajournals.org/doi/10.1161/HYPERTENSIONAHA.123.21054 10.1161/HYPERTENSIONAHA.123.21054 37170822

[63] Stowasser M, Sharman J, Leano R, Gordon RD, Ward G, Cowley D et al Evidence for Abnormal Left Ventricular Structure and Function in Normotensive Individuals with Familial Hyperaldosteronism Type I. J Clin Endocrinol Metab [Internet]. 2005 [cited 2024 Oct 9];90:5070–6. Available from: https://academic.oup.com/jcem/article-lookup/doi/10.1210/jc.2005-0681 10.1210/jc.2005-0681 15941863

[64] Stowasser M, Bachmann AW, Huggard PR, Rossetti TR, Gordon RD Severity of Hypertension in Familial Hyperaldosteronism Type I: Relationship to Gender and Degree of Biochemical Disturbance ^1^. J Clin Endocrinol Metab [Internet]. 2000 [cited 2024 Oct 9];85:2160–6. Available from: https://academic.oup.com/jcem/article-lookup/doi/10.1210/jcem.85.6.6651 10.1210/jcem.85.6.6651 10852446

[65] Mulatero P, Tizzani D, Viola A, Bertello C, Monticone S, Mengozzi G et al (2011) Prevalence and Characteristics of Familial Hyperaldosteronism: The PATOGEN Study (Primary Aldosteronism in TOrino-GENetic forms). Hypertension [Internet]. [cited 2024 Oct 9];58:797–803. Available from: https://www.ahajournals.org/doi/10.1161/HYPERTENSIONAHA.111.175083 10.1161/HYPERTENSIONAHA.111.175083 21876069

[66] Carvajal CA, Campino C, Martinez-Aguayo A, Tichauer JE, Bancalari R, Valdivia C et al A New Presentation of the Chimeric CYP11B1/CYP11B2 Gene With Low Prevalence of Primary Aldosteronism and Atypical Gene Segregation Pattern. Hypertension [Internet]. 2012 [cited 2024 Oct 9];59:85–91. Available from: https://www.ahajournals.org/doi/10.1161/HYPERTENSIONAHA.111.180513 10.1161/HYPERTENSIONAHA.111.180513 22083159

[67] Dringenberg T, Sorokina M, Ehlers M, Dekomien G, Haase M, Schulze E et al Evaluation of a Recently Established Test for Familial Hyperaldosteronism Type 1. Horm Metab Res [Internet]. 2016 [cited 2024 Oct 9];48:865–8. Available from: http://www.thieme-connect.de/DOI/DOI?10.1055/s-0042-121494 10.1055/s-0042-121494 27923252

[68] Jackson RV, Lafferty A, Torpy DJ, Stratakis C New Genetic Insights in Familial Hyperaldosteronism. Ann N Y Acad Sci [Internet]. 2002 [cited 2024 Oct 9];970:77–88. Available from: https://nyaspubs.onlinelibrary.wiley.com/doi/10.1111/j.1749-6632.2002.tb04414.x 10.1111/j.1749-6632.2002.tb04414.x 12381543

[69] Stowasser M, Wolley M, Wu A, Gordon RD, Schewe J, Stölting G et al Pathogenesis of Familial Hyperaldosteronism Type II: New Concepts Involving Anion Channels. Curr Hypertens Rep [Internet]. 2019 [cited 2024 Oct 9];21:31. Available from: http://link.springer.com/10.1007/s11906-019-0934-y 10.1007/s11906-019-0934-y 30949771

[70] Scholl UI, Stölting G, Schewe J, Thiel A, Tan H, Nelson-Williams C et al CLCN2 chloride channel mutations in familial hyperaldosteronism type II. Nat Genet [Internet]. 2018 [cited 2024 Oct 9];50:349–54. Available from: https://www.nature.com/articles/s41588-018-0048-5 10.1038/s41588-018-0048-5 PMC5862758 29403011

[71] Rege J, Nanba K, Blinder AR, Plaska S, Udager AM, Vats P et al Identification of Somatic Mutations in CLCN2 in Aldosterone-Producing Adenomas. J Endocr Soc [Internet]. 2020 [cited 2024 Oct 9];4:bvaa123. Available from: https://academic.oup.com/jes/article/doi/10.1210/jendso/bvaa123/5916595 10.1210/jendso/bvaa123 PMC7528565 33033789

[72] Scholl UI (2019) CLCN2 clicks with aldosterone-producing adenomas, too! Eur J Endocrinol [Internet]. [cited 2024 Oct 9];181:C21–2. Available from: https://academic.oup.com/ejendo/article/181/5/C21/6654057 10.1530/EJE-19-0688 31585437

[73] Schewe J, Seidel E, Forslund S, Marko L, Peters J, Muller DN et al (2019) Elevated aldosterone and blood pressure in a mouse model of familial hyperaldosteronism with ClC-2 mutation. Nat Commun [Internet]. [cited 2024 Oct 9];10:5155. Available from: https://www.nature.com/articles/s41467-019-13033-4 10.1038/s41467-019-13033-4 PMC6856192 31727896

[74] Fernandes-Rosa FL, Daniil G, Orozco IJ, Göppner C, El Zein R, Jain V et al (2018) A gain-of-function mutation in the CLCN2 chloride channel gene causes primary aldosteronism. Nat Genet [Internet]. [cited 2024 Oct 9];50:355–61. Available from: https://www.nature.com/articles/s41588-018-0053-8 10.1038/s41588-018-0053-8 29403012

[75] Sun Z-L, He J-Y, Cheng X-L, Tan X-X, Wu W-H (2022) Diagnosis, treatment and genetic analysis of a case of Familial aldosteronism type II with WFS1 gene mutation. Yi Chuan Hered 44:1072–1078 10.16288/j.yczz.22-197 36384999

[76] Pons Fernández N, Moreno F, Morata J, Moriano A, León S, De Mingo C et al (2019) Familial hyperaldosteronism type III a novel case and review of literature. Rev Endocr Metab Disord [Internet]. [cited 2024 Oct 9];20:27–36. Available from: http://link.springer.com/10.1007/s11154-018-9481-0 10.1007/s11154-018-9481-0 30569443

[77] Gomez-Sanchez CE, Oki K, Minireview Potassium Channels and Aldosterone Dysregulation: Is Primary Aldosteronism a Potassium Channelopathy? Endocrinology [Internet]. 2014 [cited 2024 Oct 9];155:47–55. Available from: https://academic.oup.com/endo/article/155/1/47/2422421 10.1210/en.2013-1733 PMC5398635 24248457

[78] Monticone S, Tetti M, Burrello J, Buffolo F, De Giovanni R, Veglio F et al Familial hyperaldosteronism type III. J Hum Hypertens [Internet]. 2017 [cited 2024 Oct 9];31:776–81. Available from: https://www.nature.com/articles/jhh201734 10.1038/jhh.2017.34 28447626

[79] Rege J, Turcu AF, Rainey WE (2020) Primary aldosteronism diagnostics: KCNJ5 mutations and hybrid steroid synthesis in aldosterone-producing adenomas. Gland Surg [Internet]. [cited 2024 Oct 9];9:3–13. Available from: http://gs.amegroups.com/article/view/34954/27987 10.21037/gs.2019.10.22 PMC7082274 32206594

[80] Choi M, Scholl UI, Yue P, Björklund P, Zhao B, Nelson-Williams C et al (2011) K ^+^ Channel Mutations in Adrenal Aldosterone-Producing Adenomas and Hereditary Hypertension. Science [Internet]. [cited 2024 Oct 9];331:768–72. Available from: https://www.science.org/doi/10.1126/science.1198785 10.1126/science.1198785 PMC3371087 21311022

[81] Corey S, Clapham DE (1998) Identification of Native Atrial G-protein-regulated Inwardly Rectifying K+ (GIRK4) Channel Homomultimers. J Biol Chem [Internet]. [cited 2024 Oct 9];273:27499–504. Available from: https://linkinghub.elsevier.com/retrieve/pii/S0021925819596971 10.1074/jbc.273.42.27499 9765280

[82] Scholl UI, Nelson-Williams C, Yue P, Grekin R, Wyatt RJ, Dillon MJ et al (2012) Hypertension with or without adrenal hyperplasia due to different inherited mutations in the potassium channel *KCNJ5*. Proc Natl Acad Sci [Internet]. [cited 2024 Oct 9];109:2533–8. Available from: 10.1073/pnas.1121407109 10.1073/pnas.1121407109 PMC3289329 22308486

[83] Kuppusamy M, Caroccia B, Stindl J, Bandulik S, Lenzini L, Gioco F et al (2014) A Novel KCNJ5-insT149 Somatic Mutation Close to, but Outside, the Selectivity Filter Causes Resistant Hypertension by Loss of Selectivity for Potassium. J Clin Endocrinol Metab [Internet]. [cited 2024 Oct 9];99:E1765–73. Available from: https://academic.oup.com/jcem/article/99/9/E1765/2537639 10.1210/jc.2014-1927 PMC4154085 25057880

[84] Oki K, Plonczynski MW, Luis Lam M, Gomez-Sanchez EP, Gomez-Sanchez CE Potassium Channel Mutant KCNJ5 T158A Expression in HAC-15 Cells Increases Aldosterone Synthesis. Endocrinology [Internet]. 2012 [cited 2024 Oct 9];153:1774–82. Available from: https://academic.oup.com/endo/article/153/4/1774/2423910 10.1210/en.2011-1733 PMC3320257 22315453

[85] Lenzini L, Rossi GP The molecular basis of primary aldosteronism: from chimeric gene to channelopathy. Curr Opin Pharmacol [Internet]. 2015 [cited 2024 Oct 9];21:35–42. Available from: https://linkinghub.elsevier.com/retrieve/pii/S1471489214001726 10.1016/j.coph.2014.12.005 25555247

[86] Tong A, Liu G, Wang F, Jiang J, Yan Z, Zhang D et al (2016) A Novel Phenotype of Familial Hyperaldosteronism Type III: Concurrence of Aldosteronism and Cushing’s Syndrome. J Clin Endocrinol Metab [Internet]. [cited 2024 Oct 9];101:4290–7. Available from: https://academic.oup.com/jcem/article/101/11/4290/2765006 10.1210/jc.2016-1504 PMC5095249 27403928

[87] Adachi M, Muroya K, Asakura Y, Sugiyama K, Homma K, Hasegawa T (2014) Discordant Genotype-Phenotype Correlation in Familial Hyperaldosteronism Type III with KCNJ5 Gene Mutation: A Patient Report and Review of the Literature. Horm Res Paediatr [Internet]. [cited 2024 Oct 9];82:138–42. Available from: https://karger.com/HRP/article/doi/10.1159/000358197 10.1159/000358197 24819081

[88] Monticone S, Bandulik S, Stindl J, Zilbermint M, Dedov I, Mulatero P et al (2015) A Case of Severe Hyperaldosteronism Caused by a De Novo Mutation Affecting a Critical Salt Bridge Kir3.4 Residue. J Clin Endocrinol Metab [Internet]. [cited 2024 Oct 9];100:E114–8. Available from: https://academic.oup.com/jcem/article-lookup/doi/10.1210/jc.2014-3636 10.1210/jc.2014-3636 PMC4283020 25322277

[89] Gomez-Sanchez CE, Qi X, Gomez-Sanchez EP, Sasano H, Bohlen MO, Wisgerhof M (2017) Disordered zonal and cellular CYP11B2 enzyme expression in familial hyperaldosteronism type 3. Mol Cell Endocrinol [Internet]. [cited 2024 Oct 9];439:74–80. Available from: https://linkinghub.elsevier.com/retrieve/pii/S0303720716304361 10.1016/j.mce.2016.10.025 PMC5123946 27793677

[90] Maria AG, Suzuki M, Berthon A, Kamilaris C, Demidowich A, Lack J et al (2020) Mosaicism for *KCNJ5* Causing Early-Onset Primary Aldosteronism due to Bilateral Adrenocortical Hyperplasia. Am J Hypertens [Internet]. [cited 2024 Oct 9];33:124–30. Available from: https://academic.oup.com/ajh/article/33/2/124/5601961 10.1093/ajh/hpz172 PMC8204147 31637427

[91] Monticone S, Hattangady NG, Penton D, Isales CM, Edwards MA, Williams TA et al (2013) A Novel Y152C KCNJ5 Mutation Responsible for Familial Hyperaldosteronism Type III. J Clin Endocrinol Metab [Internet]. [cited 2024 Oct 9];98:E1861–5. Available from: https://academic.oup.com/jcem/article-lookup/doi/10.1210/jc.2013-2428 10.1210/jc.2013-2428 PMC3816265 24037882

[92] Sertedaki A, Markou A, Vlachakis D, Kossida S, Campanac E, Hoffman DA et al (2016) Functional characterization of two novel germline mutations of the *KCNJ 5* gene in hypertensive patients without primary aldosteronism but with ACTH -dependent aldosterone hypersecretion. Clin Endocrinol (Oxf) [Internet]. [cited 2024 Oct 9];85:845–51. Available from: https://onlinelibrary.wiley.com/doi/10.1111/cen.13132 10.1111/cen.13132 PMC5118167 27293068

[93] Seidel E, Schewe J, Zhang J, Dinh HA, Forslund SK, Markó L et al (2021) Enhanced Ca ^2+^ signaling, mild primary aldosteronism, and hypertension in a familial hyperaldosteronism mouse model (*Cacna1h*^*M1560V*/+^). Proc Natl Acad Sci [Internet]. [cited 2024 Oct 9];118:e2014876118. Available from: 10.1073/pnas.2014876118 10.1073/pnas.2014876118 PMC8092574 33879608

[94] Dinh HA, Stölting G, Scholl UI (2023) CaV3.2 (CACNA1H) in Primary Aldosteronism. In: Striessnig J, editor. Volt-Gated Ca2 Channels Pharmacol Modul Their Role Hum Dis [Internet]. Cham: Springer International Publishing; [cited 2024 Oct 9]. pp. 249–62. Available from: https://link.springer.com/10.1007/164_2023_660

[95] Reimer EN, Walenda G, Seidel E, Scholl UI CACNA1HM1549V Mutant Calcium Channel Causes Autonomous Aldosterone Production in HAC15 Cells and Is Inhibited by Mibefradil. Endocrinology [Internet]. 2016 [cited 2024 Oct 9];157:3016–22. Available from: https://academic.oup.com/endo/article/157/8/3016/2422350 10.1210/en.2016-1170 27258646

[96] Nanba K, Blinder AR, Rege J, Hattangady NG, Else T, Liu C-J et al Somatic *CACNA1H* Mutation As a Cause of Aldosterone-Producing Adenoma. Hypertension [Internet]. 2020 [cited 2024 Oct 9];75:645–9. Available from: https://www.ahajournals.org/doi/10.1161/HYPERTENSIONAHA.119.14349 10.1161/HYPERTENSIONAHA.119.14349 PMC7059016 31983310

[97] Scholl UI, Stölting G, Nelson-Williams C, Vichot AA, Choi M, Loring E et al (2015) Recurrent gain of function mutation in calcium channel CACNA1H causes early-onset hypertension with primary aldosteronism. eLife [Internet]. [cited 2024 Oct 9];4:e06315. Available from: https://elifesciences.org/articles/06315 10.7554/eLife.06315 PMC4408447 25907736

[98] Daniil G, Fernandes-Rosa FL, Chemin J, Blesneac I, Beltrand J, Polak M et al CACNA1H Mutations Are Associated With Different Forms of Primary Aldosteronism. EBioMedicine [Internet]. 2016 [cited 2024 Oct 9];13:225–36. Available from: https://linkinghub.elsevier.com/retrieve/pii/S2352396416304571 10.1016/j.ebiom.2016.10.002 PMC5264314 27729216

[99] Wulczyn K, Perez-Reyes E, Nussbaum RL, Park M Primary aldosteronism associated with a germline variant in *CACNA1H*. BMJ Case Rep [Internet]. 2019 [cited 2024 Oct 9];12:e229031. Available from: https://casereports.bmj.com/lookup/doi/10.1136/bcr-2018-229031 10.1136/bcr-2018-229031 PMC6536178 31126930

[100] Monticone S, Buffolo F, Tetti M, Veglio F, Pasini B, Mulatero P (2018) GENETICS IN ENDOCRINOLOGY: The expanding genetic horizon of primary aldosteronism. Eur J Endocrinol [Internet]. [cited 2024 Oct 9];178:R101–11. Available from: https://academic.oup.com/ejendo/article/178/3/R101/6655260 10.1530/EJE-17-0946 29348113

[101] Pinggera A, Mackenroth L, Rump A, Schallner J, Beleggia F, Wollnik B et al New gain-of-function mutation shows CACNA1D as recurrently mutated gene in autism spectrum disorders and epilepsy. Hum Mol Genet [Internet]. 2017 [cited 2024 Oct 9];26:2923–32. Available from: https://academic.oup.com/hmg/article/26/15/2923/3797161 10.1093/hmg/ddx175 PMC5886262 28472301

[102] Ezell KM, Tinker RJ, Furuta Y, Gulsevin A, Bastarache L, Hamid R et al (2024) Undiagnosed Disease Network collaborative approach in diagnosing rare disease in a patient with a mosaic *CACNA1D* variant. Am J Med Genet A [Internet]. [cited 2024 Oct 9];194:e63597. Available from: https://onlinelibrary.wiley.com/doi/10.1002/ajmg.a.63597 10.1002/ajmg.a.63597 PMC11161305 38511854

[103] De Mingo Alemany MC, Mifsud Grau L, Moreno Macián F, Ferrer Lorente B, León Cariñena S A de novo CACNA1D missense mutation in a patient with congenital hyperinsulinism, primary hyperaldosteronism and hypotonia. Channels [Internet]. 2020 [cited 2024 Oct 9];14:175–80. Available from: https://www.tandfonline.com/doi/full/10.1080/19336950.2020.1761171 10.1080/19336950.2020.1761171 PMC7219433 32336187

[104] Scholl UI, Goh G, Stölting G, De Oliveira RC, Choi M, Overton JD et al (2013) Somatic and germline CACNA1D calcium channel mutations in aldosterone-producing adenomas and primary aldosteronism. Nat Genet [Internet]. [cited 2024 Oct 9];45:1050–4. Available from: https://www.nature.com/articles/ng.2695 10.1038/ng.2695 PMC3876926 23913001

[105] Azizan EAB, Poulsen H, Tuluc P, Zhou J, Clausen MV, Lieb A et al (2013) Somatic mutations in ATP1A1 and CACNA1D underlie a common subtype of adrenal hypertension. Nat Genet [Internet]. [cited 2024 Oct 9];45:1055–60. Available from: https://www.nature.com/articles/ng.2716 10.1038/ng.2716 23913004

[106] Zennaro M-C, Jeunemaitre X (2016) SFE/SFHTA/AFCE consensus on primary aldosteronism, part 5: Genetic diagnosis of primary aldosteronism. Ann Endocrinol [Internet]. [cited 2024 Oct 9];77:214–9. Available from: https://linkinghub.elsevier.com/retrieve/pii/S000342661630021X 10.1016/j.ando.2016.02.006 27315758

[107] Semenova NA, Ryzhkova OR, Strokova TV, Taran NN The third case report a patient with primary aldosteronism, seizures, and neurologic abnormalities (PASNA) syndrome de novo variant mutations in the CACNA1D gene. Zhurnal Nevrol Psikhiatrii Im SS Korsakova [Internet]. 2018 [cited 2024 Oct 9];118:49. Available from: http://www.mediasphera.ru/issues/zhurnal-nevrologii-i-psikhiatrii-im-s-s-korsakova/2018/12/downloads/ru/1199772982018121049 10.17116/jnevro201811812149 30698561

[108] Stölting G, Dinh HA, Volkert M, Hellmig N, Schewe J, Hennicke L et al (2023) Isradipine therapy in Cacna1dIle772Met/+ mice ameliorates primary aldosteronism and neurologic abnormalities. JCI Insight [Internet]. [cited 2024 Oct 9];8:e162468. Available from: https://insight.jci.org/articles/view/162468 10.1172/jci.insight.162468 PMC10619505 37698934

[109] Gadelha M, Gatto F, Wildemberg LE, Fleseriu M (2023) Cushing’s syndrome. The Lancet [Internet]. [cited 2024 Oct 14];402:2237–52. Available from: https://linkinghub.elsevier.com/retrieve/pii/S014067362301961X 10.1016/S0140-6736(23)01961-X 37984386

[110] Reincke M, Fleseriu M, Cushing Syndrome (2023) A Review. JAMA [Internet]. [cited 2024 Oct 14];330:170. Available from: https://jamanetwork.com/journals/jama/fullarticle/2807073 10.1001/jama.2023.11305 37432427

[111] Newell-Price J, Bertagna X, Grossman AB, Nieman LK (2006) Cushing’s syndrome. The Lancet [Internet]. [cited 2024 Oct 14];367:1605–17. Available from: https://linkinghub.elsevier.com/retrieve/pii/S0140673606686996 10.1016/S0140-6736(06)68699-6 16698415

[112] Lacroix A, Feelders RA, Stratakis CA, Nieman LK (2015) Cushing’s syndrome. The Lancet [Internet]. [cited 2024 Oct 14];386:913–27. Available from: https://linkinghub.elsevier.com/retrieve/pii/S0140673614613751 10.1016/S0140-6736(14)61375-1 26004339

[113] Savage MO, Ferrigno R (2024) Paediatric Cushing’s disease: long-term outcome and predictors of recurrence. Front Endocrinol [Internet]. [cited 2024 Oct 14];15:1345174. Available from: https://www.frontiersin.org/articles/10.3389/fendo.2024.1345174/full 10.3389/fendo.2024.1345174 PMC10838966 38318299

[114] Ferrigno R, Hasenmajer V, Caiulo S, Minnetti M, Mazzotta P, Storr HL et al Paediatric Cushing’s disease: Epidemiology, pathogenesis, clinical management and outcome. Rev Endocr Metab Disord [Internet]. 2021 [cited 2024 Oct 14];22:817–35. Available from: https://link.springer.com/10.1007/s11154-021-09626-4 10.1007/s11154-021-09626-4 PMC8724222 33515368

[115] Isidori AM, Kaltsas GA, Grossman AB (2006) Ectopic ACTH Syndrome. In: Arzt E, Bronstein M, Guitelman M, editors. Front Horm Res [Internet]. Basel: KARGER; [cited 2024 Oct 14]. pp. 143–56. Available from: https://karger.com/books/book/2923/chapter/5819715 10.1159/000094323 16809930

[116] Lodish MB, Keil MF, Stratakis CA Cushing’s Syndrome in Pediatrics. Endocrinol Metab Clin North Am [Internet]. 2018 [cited 2024 Oct 14];47:451–62. Available from: https://linkinghub.elsevier.com/retrieve/pii/S0889852918300161 10.1016/j.ecl.2018.02.008 PMC5962291 29754644

[117] Lodish M, Stratakis CA A genetic and molecular update on adrenocortical causes of Cushing syndrome. Nat Rev Endocrinol [Internet]. 2016 [cited 2024 Oct 14];12:255–62. Available from: https://www.nature.com/articles/nrendo.2016.24 10.1038/nrendo.2016.24 26965378

[118] Albani A, Theodoropoulou M, Reincke M Genetics of Cushing’s disease. Clin Endocrinol (Oxf) [Internet]. 2018 [cited 2024 Oct 14];88:3–12. Available from: https://onlinelibrary.wiley.com/doi/10.1111/cen.13457 10.1111/cen.13457 28850717

[119] Brown RJ, Kelly MH, Collins MT Cushing Syndrome in the McCune-Albright Syndrome. J Clin Endocrinol Metab [Internet]. 2010 [cited 2024 Oct 14];95:1508–15. Available from: https://academic.oup.com/jcem/article/95/4/1508/2596338 10.1210/jc.2009-2321 PMC2853983 20157193

[120] Carney JA, Young WF, Stratakis CA (2011) Primary Bimorphic Adrenocortical Disease: Cause of Hypercortisolism in McCune-Albright Syndrome. Am J Surg Pathol [Internet]. [cited 2024 Oct 14];35:1311–26. Available from: https://journals.lww.com/00000478-201109000-00007 10.1097/PAS.0b013e31821ec4ce PMC4140081 21836496

[121] Zhang CD, Pichurin PN, Bobr A, Lyden ML, Young WF, Bancos I Cushing syndrome: uncovering Carney complex due to novel PRKAR1A mutation. Endocrinol Diabetes Metab Case Rep [Internet]. 2019 [cited 2024 Oct 14];2019. Available from: https://edm.bioscientifica.com/view/journals/edm/2019/1/EDM18-0150.xml 10.1530/EDM-18-0150 PMC6432981 30897549

[122] Cavalcante IP, Berthon A, Fragoso MC, Reincke M, Stratakis CA, Ragazzon B et al (2022) Primary bilateral macronodular adrenal hyperplasia: definitely a genetic disease. Nat Rev Endocrinol [Internet]. [cited 2024 Oct 14];18:699–711. Available from: https://www.nature.com/articles/s41574-022-00718-y 10.1038/s41574-022-00718-y 35922573

[123] Bouys L, Chiodini I, Arlt W, Reincke M, Bertherat J Update on primary bilateral macronodular adrenal hyperplasia (PBMAH). Endocrine [Internet]. 2021 [cited 2024 Oct 14];71:595–603. Available from: http://link.springer.com/10.1007/s12020-021-02645-w 10.1007/s12020-021-02645-w 33587256

[124] Araujo-Castro M, Reincke M Primary bilateral macronodular adrenal hyperplasia: A series of 32 cases and literature review. Endocrinol Diabetes Nutr Engl Ed [Internet]. 2023 [cited 2024 Oct 14];70:229–39. Available from: https://linkinghub.elsevier.com/retrieve/pii/S2530018023000720 10.1016/j.endien.2023.04.005 37116968

[125] Thakker RV, Newey PJ, Walls GV, Bilezikian J, Dralle H, Ebeling PR et al Clinical Practice Guidelines for Multiple Endocrine Neoplasia Type 1 (MEN1). J Clin Endocrinol Metab [Internet]. 2012 [cited 2024 Oct 14];97:2990–3011. Available from: https://academic.oup.com/jcem/article/97/9/2990/2536740 10.1210/jc.2012-1230 22723327

[126] Faucz FR, Tirosh A, Tatsi C, Berthon A, Hernández-Ramírez LC, Settas N et al (2017) Somatic USP8 Gene Mutations Are a Common Cause of Pediatric Cushing Disease. J Clin Endocrinol Metab [Internet]. [cited 2024 Oct 14];102:2836–43. Available from: http://academic.oup.com/jcem/article/102/8/2836/3819476/Somatic-USP8-Gene-Mutations-Are-a-Common-Cause-of 10.1210/jc.2017-00161 PMC5546857 28505279

[127] Perez-Rivas LG, Theodoropoulou M, Ferraù F, Nusser C, Kawaguchi K, Stratakis CA et al (2015) The Gene of the Ubiquitin-Specific Protease 8 Is Frequently Mutated in Adenomas Causing Cushing’s Disease. J Clin Endocrinol Metab [Internet]. [cited 2024 Oct 14];100:E997–1004. Available from: https://academic.oup.com/jcem/article-lookup/doi/10.1210/jc.2015-1453 10.1210/jc.2015-1453 PMC4490309 25942478

[128] Fassnacht M, Dekkers OM, Else T, Baudin E, Berruti A, De Krijger RR et al (2018) European Society of Endocrinology Clinical Practice Guidelines on the management of adrenocortical carcinoma in adults, in collaboration with the European Network for the Study of Adrenal Tumors. Eur J Endocrinol [Internet]. [cited 2024 Oct 14];179:G1–46. Available from: https://academic.oup.com/ejendo/article/179/4/G1/6655445 10.1530/EJE-18-0608 30299884

[129] Wasserman JD, Novokmet A, Eichler-Jonsson C, Ribeiro RC, Rodriguez-Galindo C, Zambetti GP et al (2015) Prevalence and Functional Consequence of *TP53* Mutations in Pediatric Adrenocortical Carcinoma: A Children’s Oncology Group Study. J Clin Oncol [Internet]. [cited 2024 Oct 14];33:602–9. Available from: 10.1200/JCO.2013.52.6863 10.1200/JCO.2013.52.6863 PMC4517369 25584008

[130] Domènech M, Grau E, Solanes A, Izquierdo A, Del Valle J, Carrato C et al Characteristics of Adrenocortical Carcinoma Associated With Lynch Syndrome. J Clin Endocrinol Metab [Internet]. 2021 [cited 2024 Oct 14];106:318–25. Available from: https://academic.oup.com/jcem/article/106/2/318/5981336 10.1210/clinem/dgaa833 33185660

[131] Flynn JT, Kaelber DC, Baker-Smith CM, Blowey D, Carroll AE, Daniels SR et al Clinical Practice Guideline for Screening and Management of High Blood Pressure in Children and Adolescents. Pediatrics [Internet]. 2017 [cited 2024 Oct 14];140:e20171904. Available from: https://publications.aap.org/pediatrics/article/140/3/e20171904/38358/Clinical-Practice-Guideline-for-Screening-and 10.1542/peds.2017-1904 28827377

[132] Fallo F, Di Dalmazi G, Beuschlein F, Biermasz NR, Castinetti F, Elenkova A et al (2022) Diagnosis and management of hypertension in patients with Cushing’s syndrome: a position statement and consensus of the Working Group on Endocrine Hypertension of the European Society of Hypertension. J Hypertens [Internet]. [cited 2024 Oct 14];40:2085–101. Available from: https://journals.lww.com/10.1097/HJH.0000000000003252 10.1097/HJH.0000000000003252 35950979

[133] Lodish MB, Sinaii N, Patronas N, Batista DL, Keil M, Samuel J et al (2009) Blood Pressure in Pediatric Patients with Cushing Syndrome. J Clin Endocrinol Metab [Internet]. [cited 2024 Oct 14];94:2002–8. Available from: https://academic.oup.com/jcem/article/94/6/2002/2596878 10.1210/jc.2008-2694 PMC2690429 19293264

[134] Magiakou MA, Mastorakos G, Zachman K, Chrousos GP (1997) Blood Pressure in Children and Adolescents with Cushing’s Syndrome before and after Surgical Cure. J Clin Endocrinol Metab [Internet]. [cited 2024 Oct 14];82:1734–8. Available from: https://academic.oup.com/jcem/article/82/6/1734/2656372 10.1210/jcem.82.6.3985 9177372

[135] Ferrari P, Krozowski Z (2000) Role of the 11β-hydroxysteroid dehydrogenase type 2 in blood pressure regulation. Kidney Int [Internet]. [cited 2024 Oct 14];57:1374–81. Available from: https://linkinghub.elsevier.com/retrieve/pii/S008525381546884X 10.1046/j.1523-1755.2000.00978.x 10760070

[136] Barbot M, Ceccato F, Scaroni C (2019) The Pathophysiology and Treatment of Hypertension in Patients With Cushing’s Syndrome. Front Endocrinol [Internet]. [cited 2024 Oct 14];10:321. Available from: https://www.frontiersin.org/article/10.3389/fendo.2019.00321/full 10.3389/fendo.2019.00321 PMC6536607 31164868

[137] Baid S, Nieman LK (2004) Glucocorticoid excess and hypertension. Curr Hypertens Rep [Internet]. [cited 2024 Oct 14];6:493–9. Available from: http://link.springer.com/10.1007/s11906-004-0046-0 10.1007/s11906-004-0046-0 15527696

[138] Quinkler M, Stewart PM (2003) Hypertension and the Cortisol-Cortisone Shuttle. J Clin Endocrinol Metab [Internet]. [cited 2024 Oct 14];88:2384–92. Available from: https://academic.oup.com/jcem/article-lookup/doi/10.1210/jc.2003-030138 10.1210/jc.2003-030138 12788832

[139] Isidori AM, Graziadio C, Paragliola RM, Cozzolino A, Ambrogio AG, Colao A et al (2015) The hypertension of Cushing’s syndrome: controversies in the pathophysiology and focus on cardiovascular complications. J Hypertens [Internet]. [cited 2024 Oct 14];33:44–60. Available from: https://journals.lww.com/00004872-201501000-00006 10.1097/HJH.0000000000000415 PMC4342316 25415766

[140] Tarçın G, Çatlı G, Çetinkaya S, Eren E, Kardelen AD, Akıncı A et al Clinical features, diagnosis and treatment outcomes of Cushing’s disease in children: A multicenter study. Clin Endocrinol (Oxf) [Internet]. 2024 [cited 2024 Oct 14];100:19–28. Available from: https://onlinelibrary.wiley.com/doi/10.1111/cen.14980 10.1111/cen.14980 37814958

[141] Batista DL, Riar J, Keil M, Stratakis CA Diagnostic Tests for Children Who Are Referred for the Investigation of Cushing Syndrome. Pediatrics [Internet]. 2007 [cited 2024 Oct 14];120:e575–86. Available from: https://publications.aap.org/pediatrics/article/120/3/e575/71160/Diagnostic-Tests-for-Children-Who-Are-Referred-for 10.1542/peds.2006-2402 17698579

[142] Fleseriu M, Auchus R, Bancos I, Ben-Shlomo A, Bertherat J, Biermasz NR et al (2021) Consensus on diagnosis and management of Cushing’s disease: a guideline update. Lancet Diabetes Endocrinol [Internet]. [cited 2024 Oct 14];9:847–75. Available from: https://linkinghub.elsevier.com/retrieve/pii/S2213858721002357 10.1016/S2213-8587(21)00235-7 PMC8743006 34687601

[143] Batista D, Gennari M, Riar J, Chang R, Keil MF, Oldfield EH et al (2006) An Assessment of Petrosal Sinus Sampling for Localization of Pituitary Microadenomas in Children with Cushing Disease. J Clin Endocrinol Metab [Internet]. [cited 2024 Oct 14];91:221–4. Available from: https://academic.oup.com/jcem/article-lookup/doi/10.1210/jc.2005-1096 10.1210/jc.2005-1096 16219718

[144] Storr HL, Alexandraki KI, Martin L, Isidori AM, Kaltsas GA, Monson JP et al (2011) Comparisons in the epidemiology, diagnostic features and cure rate by transsphenoidal surgery between paediatric and adult-onset Cushing’s disease. Eur J Endocrinol [Internet]. [cited 2024 Oct 14];164:667–74. Available from: https://academic.oup.com/ejendo/article/164/5/667/6676937 10.1530/EJE-10-1120 21310872

[145] Minnetti M, Caiulo S, Ferrigno R, Baldini-Ferroli B, Bottaro G, Gianfrilli D et al (2020) Abnormal linear growth in paediatric adrenal diseases: Pathogenesis, prevalence and management. Clin Endocrinol (Oxf) [Internet]. [cited 2024 Oct 14];92:98–108. Available from: https://onlinelibrary.wiley.com/doi/10.1111/cen.14131 10.1111/cen.14131 31747461

[146] Dupuis CC, Storr HL, Perry LA, Ho JTF, Ahmed L, Ong KK et al Abnormal puberty in paediatric Cushing’s disease: relationship with adrenal androgen, sex hormone binding globulin and gonadotrophin concentrations. Clin Endocrinol (Oxf) [Internet]. 2007 [cited 2024 Oct 14];66:838–43. Available from: https://onlinelibrary.wiley.com/doi/10.1111/j.1365-2265.2007.02822.x 10.1111/j.1365-2265.2007.02822.x 17437509

[147] Yordanova G, Martin L, Afshar F, Sabin I, Alusi G, Plowman NP et al Long-term outcomes of children treated for Cushing’s disease: a single center experience. Pituitary [Internet]. 2016 [cited 2024 Oct 14];19:612–24. Available from: http://link.springer.com/10.1007/s11102-016-0756-8 10.1007/s11102-016-0756-8 PMC5080319 27678103

[148] Lonser RR, Wind JJ, Nieman LK, Weil RJ, DeVroom HL, Oldfield EH Outcome of Surgical Treatment of 200 Children With Cushing’s Disease. J Clin Endocrinol Metab [Internet]. 2013 [cited 2024 Oct 14];98:892–901. Available from: https://academic.oup.com/jcem/article/98/3/892/2536508 10.1210/jc.2012-3604 PMC3590477 23372173

[149] Storr HL, Plowman PN, Carroll PV, François I, Krassas GE, Afshar F et al (2003) Clinical and Endocrine Responses to Pituitary Radiotherapy in Pediatric Cushing’s Disease: An Effective Second-Line Treatment. J Clin Endocrinol Metab [Internet]. [cited 2024 Oct 14];88:34–7. Available from: https://academic.oup.com/jcem/article-lookup/doi/10.1210/jc.2002-021032 10.1210/jc.2002-021032 12519825

[150] Shrivastava A, Mohammed N, Xu Z, Liščák R, Kosak M, Krsek M et al (2019) Outcomes After Gamma Knife Stereotactic Radiosurgery in Pediatric Patients with Cushing Disease or Acromegaly: A Multi-Institutional Study. World Neurosurg [Internet]. [cited 2024 Oct 14];125:e1104–13. Available from: https://linkinghub.elsevier.com/retrieve/pii/S1878875019303705 10.1016/j.wneu.2019.01.252 30790739

[151] Pivonello R, De Leo M, Cozzolino A, Colao A (2015) The Treatment of Cushing’s Disease. Endocr Rev [Internet]. [cited 2024 Oct 14];36:385–486. Available from: https://academic.oup.com/edrv/article/36/4/385/2354703 10.1210/er.2013-1048 PMC4523083 26067718

[152] Motte E, Rothenbuhler A, Gaillard S, Lahlou N, Teinturier C, Coutant R et al (2018) Mitotane (op’DDD) restores growth and puberty in nine children with Cushing’s disease. Endocr Connect [Internet]. [cited 2024 Oct 14];7:1280–7. Available from: https://ec.bioscientifica.com/view/journals/ec/7/12/EC-18-0215.xml 10.1530/EC-18-0215 PMC6240149 30352417

[153] Riedmeier M, Antonini SRR, Brandalise S, Costa TEJB, Daiggi CM, De Figueiredo BC et al (2024) International consensus on mitotane treatment in pediatric patients with adrenal cortical tumors: indications, therapy, and management of adverse effects. Eur J Endocrinol [Internet]. [cited 2024 Oct 14];190:G15–24. Available from: https://academic.oup.com/ejendo/article/190/4/G15/7637361 10.1093/ejendo/lvae038 38552173

[154] Pivonello R, Fleseriu M, Newell-Price J, Bertagna X, Findling J, Shimatsu A et al (2020) Efficacy and safety of osilodrostat in patients with Cushing’s disease (LINC 3): a multicentre phase III study with a double-blind, randomised withdrawal phase. Lancet Diabetes Endocrinol [Internet]. [cited 2024 Oct 14];8:748–61. Available from: https://linkinghub.elsevier.com/retrieve/pii/S2213858720302400 10.1016/S2213-8587(20)30240-0 32730798

[155] Fassnacht M, Tsagarakis S, Terzolo M, Tabarin A, Sahdev A, Newell-Price J et al (2023) European Society of Endocrinology clinical practice guidelines on the management of adrenal incidentalomas, in collaboration with the European Network for the Study of Adrenal Tumors. Eur J Endocrinol [Internet]. [cited 2024 Oct 14];189:G1–42. Available from: https://academic.oup.com/ejendo/article/189/1/G1/7198474 10.1093/ejendo/lvad066 37318239

[156] Karageorgiadis AS, Papadakis GZ, Biro J, Keil MF, Lyssikatos C, Quezado MM et al (2015) Ectopic Adrenocorticotropic Hormone and Corticotropin-Releasing Hormone Co-Secreting Tumors in Children and Adolescents Causing Cushing Syndrome: A Diagnostic Dilemma and How to Solve It. J Clin Endocrinol Metab [Internet]. [cited 2024 Oct 14];100:141–8. Available from: https://academic.oup.com/jcem/article-lookup/doi/10.1210/jc.2014-2945 10.1210/jc.2014-2945 PMC4283025 25291050

[157] More J, Young J, Reznik Y, Raverot G, Borson-Chazot F, Rohmer V et al (2011) Ectopic ACTH Syndrome in Children and Adolescents. J Clin Endocrinol Metab [Internet]. [cited 2024 Oct 14];96:1213–22. Available from: https://academic.oup.com/jcem/article-lookup/doi/10.1210/jc.2010-2276 10.1210/jc.2010-2276 21346064

[158] Reincke M, Albani A, Assie G, Bancos I, Brue T, Buchfelder M et al (2021) Corticotroph tumor progression after bilateral adrenalectomy (Nelson’s syndrome): systematic review and expert consensus recommendations. Eur J Endocrinol [Internet]. [cited 2024 Oct 14];184:P1–16. Available from: https://academic.oup.com/ejendo/article/184/3/P1/6653849 10.1530/EJE-20-1088 PMC8060870 33444221

[159] Thomas CGJ, Smith AT, Benson M, Griffith J (1984) Nelson’s syndrome after cushing’s disease in childhood: a continuing problem. Surgery 96:1067–1077 6505960

[160] Pacak K, Eisenhofer G, Ahlman H, Bornstein SR, Gimenez-Roqueplo A-P, Grossman AB et al (2007) Pheochromocytoma: recommendations for clinical practice from the First International Symposium. Nat Clin Pract Endocrinol Metab [Internet]. [cited 2024 Oct 15];3:92–102. Available from: https://www.nature.com/articles/ncpendmet0396 10.1038/ncpendmet0396 17237836

[161] Lenders JWM, Duh Q-Y, Eisenhofer G, Gimenez-Roqueplo A-P, Grebe SKG, Murad MH et al (2014) Pheochromocytoma and Paraganglioma: An Endocrine Society Clinical Practice Guideline. J Clin Endocrinol Metab [Internet]. [cited 2024 Oct 15];99:1915–42. Available from: https://academic.oup.com/jcem/article/99/6/1915/2537399 10.1210/jc.2014-1498 24893135

[162] Pacak K, Linehan WM, Eisenhofer G, Walther MM, Goldstein DS (2001) Recent Advances in Genetics, Diagnosis, Localization, and Treatment of Pheochromocytoma. Ann Intern Med [Internet]. [cited 2024 Oct 15];134:315. Available from: http://annals.org/article.aspx?doi=10.7326/0003-4819-134-4-200102200-00016 10.7326/0003-4819-134-4-200102200-00016 11182843

[163] Stein PP, Black HR (1991) A Simplified Diagnostic Approach to Pheochromocytoma A Review of the Literature and Report of One Institutionʼs Experience: Medicine (Baltimore) [Internet]. [cited 2024 Oct 15];70:46–66. Available from: http://journals.lww.com/00005792-199101000-00004 10.1097/00005792-199101000-00004 1988766

[164] Gimenez-Roqueplo A-P, Dahia P, Robledo M (2012) An Update on the Genetics of Paraganglioma, Pheochromocytoma, and Associated Hereditary Syndromes. Horm Metab Res [Internet]. [cited 2024 Oct 15];44:328–33. Available from: http://www.thieme-connect.de/DOI/DOI?10.1055/s-0031-1301302 10.1055/s-0031-1301302 22328163

[165] Neumann HPH, Bausch B, McWhinney SR, Bender BU, Gimm O, Franke G et al (2002) Germ-Line Mutations in Nonsyndromic Pheochromocytoma. N Engl J Med [Internet]. [cited 2024 Oct 15];346:1459–66. Available from: http://www.nejm.org/doi/abs/10.1056/NEJMoa020152 10.1056/NEJMoa020152 12000816

[166] Crona J, Delgado Verdugo A, Maharjan R, Stålberg P, Granberg D, Hellman P et al (2013) Somatic Mutations in H-RAS in Sporadic Pheochromocytoma and Paraganglioma Identified by Exome Sequencing. J Clin Endocrinol Metab [Internet]. [cited 2024 Oct 15];98:E1266–71. Available from: https://academic.oup.com/jcem/article/98/7/E1266/2536831 10.1210/jc.2012-4257 23640968

[167] López-Jiménez E, Gómez-López G, Leandro-García LJ, Muñoz I, Schiavi F, Montero-Conde C et al Research Resource: Transcriptional Profiling Reveals Different Pseudohypoxic Signatures in SDHB and VHL-Related Pheochromocytomas. Mol Endocrinol [Internet]. 2010 [cited 2024 Oct 15];24:2382–91. Available from: https://academic.oup.com/mend/article/24/12/2382/2738107 10.1210/me.2010-0256 PMC5417372 20980436

[168] Favier J, Gimenez-Roqueplo A-P, Pheochromocytomas The (pseudo)-hypoxia hypothesis. Best Pract Res Clin Endocrinol Metab [Internet]. 2010 [cited 2024 Oct 15];24:957–68. Available from: https://linkinghub.elsevier.com/retrieve/pii/S1521690X10001314 10.1016/j.beem.2010.10.004 21115164

[169] Jochmanova I, Yang C, Zhuang Z, Pacak K (2013) Hypoxia-Inducible Factor Signaling in Pheochromocytoma: Turning the Rudder in the Right Direction. JNCI J Natl Cancer Inst [Internet]. [cited 2024 Oct 15];105:1270–83. Available from: https://academic.oup.com/jnci/article-lookup/doi/10.1093/jnci/djt201 10.1093/jnci/djt201 PMC3888279 23940289

[170] Stackpole RH, Melicow MM, Uson AC (1963) Pheochromocytoma in children. J Pediatr [Internet]. [cited 2024 Oct 15];63:315–30. Available from: https://linkinghub.elsevier.com/retrieve/pii/S0022347663803455 10.1016/s0022-3476(63)80345-5 14046640

[171] Pamporaki C, Hamplova B, Peitzsch M, Prejbisz A, Beuschlein F, Timmers HJLM et al Characteristics of Pediatric vs Adult Pheochromocytomas and Paragangliomas. J Clin Endocrinol Metab [Internet]. 2017 [cited 2024 Oct 15];102:1122–32. Available from: https://academic.oup.com/jcem/article-lookup/doi/10.1210/jc.2016-3829 10.1210/jc.2016-3829 PMC5460722 28324046

[172] Kaufman BH, Telander RL, Van Heerden JA, Zimmerman D, Sheps SG, Dawson B Pheochromocytoma in the pediatric age group: Current status. J Pediatr Surg [Internet]. 1983 [cited 2024 Oct 15];18:879–84. Available from: https://linkinghub.elsevier.com/retrieve/pii/S0022346883800402 10.1016/s0022-3468(83)80040-2 6141233

[173] Eisenhofer G, Timmers HJ, Lenders JWM, Bornstein SR, Tiebel O, Mannelli M et al (2011) Age at Diagnosis of Pheochromocytoma Differs According to Catecholamine Phenotype and Tumor Location. J Clin Endocrinol Metab [Internet]. [cited 2024 Oct 15];96:375–84. Available from: https://academic.oup.com/jcem/article-lookup/doi/10.1210/jc.2010-1588 10.1210/jc.2010-1588 PMC3048320 21147885

[174] Casey RT, Hendriks E, Deal C, Waguespack SG, Wiegering V, Redlich A et al (2024) International consensus statement on the diagnosis and management of phaeochromocytoma and paraganglioma in children and adolescents. Nat Rev Endocrinol [Internet]. [cited 2024 Nov 17];20:729–48. Available from: https://www.nature.com/articles/s41574-024-01024-5 10.1038/s41574-024-01024-5 39147856

[175] Lenders JWM, Pacak K, Walther MM, Linehan WM, Mannelli M, Friberg P et al (2002) Biochemical Diagnosis of Pheochromocytoma: Which Test Is Best? JAMA [Internet]. [cited 2024 Oct 15];287. Available from: http://jama.jamanetwork.com/article.aspx?doi=10.1001/jama.287.11.1427 10.1001/jama.287.11.1427 11903030

[176] Chen H, Sippel RS, O’Dorisio MS, Vinik AI, Lloyd RV, Pacak K The North American Neuroendocrine Tumor Society Consensus Guideline for the Diagnosis and Management of Neuroendocrine Tumors: Pheochromocytoma, Paraganglioma, and Medullary Thyroid Cancer. Pancreas [Internet]. 2010 [cited 2024 Oct 15];39:775–83. Available from: https://journals.lww.com/00006676-201008000-00007 10.1097/MPA.0b013e3181ebb4f0 PMC3419007 20664475

[177] Brain KL, Kay J, Shine B (2006) Measurement of Urinary Metanephrines to Screen for Pheochromocytoma in an Unselected Hospital Referral Population. Clin Chem [Internet]. [cited 2024 Oct 15];52:2060–4. Available from: https://academic.oup.com/clinchem/article/52/11/2060/5626788 10.1373/clinchem.2006.070805 PMC2640466 16990424

[178] Taïeb D, Pacak K Molecular imaging and theranostic approaches in pheochromocytoma and paraganglioma. Cell Tissue Res [Internet]. 2018 [cited 2024 Oct 15];372:393–401. Available from: http://link.springer.com/10.1007/s00441-018-2791-4 10.1007/s00441-018-2791-4 PMC7442158 29450723

[179] Krokhmal AA, Kwatra N, Drubach L, Weldon CB, Janeway KA, DuBois SG et al (2022) ^68^ Ga-DOTATATE PET and functional imaging in pediatric pheochromocytoma and paraganglioma. Pediatr Blood Cancer [Internet]. [cited 2024 Oct 15];69:e29740. Available from: https://onlinelibrary.wiley.com/doi/10.1002/pbc.29740 10.1002/pbc.29740 35484995

[180] Januszewicz P, Wieteska-Klimczak A, Wyszyńska T Pheochromocytoma in Children: Difficulties in Diagnosis and Localization. Clin Exp Hypertens A [Internet]. 1990 [cited 2024 Oct 15];12:571–9. Available from: http://www.tandfonline.com/doi/full/10.3109/10641969009073485 10.3109/10641969009073485 2196128

[181] Pham TH, Moir C, Thompson GB, Zarroug AE, Hamner CE, Farley D et al Pheochromocytoma and Paraganglioma in Children: A Review of Medical and Surgical Management at a Tertiary Care Center. Pediatrics [Internet]. 2006 [cited 2024 Oct 15];118:1109–17. Available from: https://publications.aap.org/pediatrics/article/118/3/1109/69394/Pheochromocytoma-and-Paraganglioma-in-Children-A 10.1542/peds.2005-2299 16951005

[182] Ciftci AO, Tanyel FC, Şenocak ME, Büyükpamukçu N Pheochromocytoma in children. J Pediatr Surg [Internet]. 2001 [cited 2024 Oct 15];36:447–52. Available from: https://linkinghub.elsevier.com/retrieve/pii/S0022346801529917 10.1053/jpsu.2001.21612 11226993

[183] Young WF, Calhoun DA, Lenders JWM, Stowasser M, Textor SC Screening for Endocrine Hypertension: An Endocrine Society Scientific Statement. Endocr Rev [Internet]. 2017 [cited 2024 Oct 15];38:103–22. Available from: https://academic.oup.com/edrv/article/38/2/103/3104343

[184] Weingarten TN, Welch TL, Moore TL, Walters GF, Whipple JL, Cavalcante A et al (2017) Preoperative Levels of Catecholamines and Metanephrines and Intraoperative Hemodynamics of Patients Undergoing Pheochromocytoma and Paraganglioma Resection. Urology [Internet]. [cited 2024 Oct 15];100:131–8. Available from: https://linkinghub.elsevier.com/retrieve/pii/S0090429516307191 10.1016/j.urology.2016.10.012 27769919

[185] Pacak K (2007) Preoperative Management of the Pheochromocytoma Patient. J Clin Endocrinol Metab [Internet]. [cited 2024 Oct 15];92:4069–79. Available from: https://academic.oup.com/jcem/article/92/11/4069/2597882 10.1210/jc.2007-1720 17989126

[186] Neumann HPH, Young WF, Eng C (2019) Pheochromocytoma and Paraganglioma. Longo DL, editor. N Engl J Med [Internet]. [cited 2024 Oct 15];381:552–65. Available from: http://www.nejm.org/doi/10.1056/NEJMra1806651 10.1056/NEJMra1806651 31390501

[187] Kohlenberg J, Welch B, Hamidi O, Callstrom M, Morris J, Sprung J et al (2019) Efficacy and Safety of Ablative Therapy in the Treatment of Patients with Metastatic Pheochromocytoma and Paraganglioma. Cancers [Internet]. [cited 2024 Oct 15];11:195. Available from: https://www.mdpi.com/2072-6694/11/2/195 10.3390/cancers11020195 PMC6407137 30736463

[188] Young WF Metastatic Pheochromocytoma: In Search of a Cure. Endocrinology [Internet]. 2020 [cited 2024 Oct 15];161:bqz019. Available from: https://academic.oup.com/endo/article/doi/10.1210/endocr/bqz019/5775763 10.1210/endocr/bqz019 32126137

[189] Yau M, Haider S, Khattab A, Ling C, Mathew M, Zaidi S et al (2017) Clinical, genetic, and structural basis of apparent mineralocorticoid excess due to 11β-hydroxysteroid dehydrogenase type 2 deficiency. Proc Natl Acad Sci [Internet]. [cited 2024 Oct 15];114. Available from: 10.1073/pnas.1716621115 10.1073/pnas.1716621115 PMC5748222 29229831

[190] Fan P, Lu Y-T, Yang K-Q, Zhang D, Liu X-Y, Tian T et al (2020) Apparent mineralocorticoid excess caused by novel compound heterozygous mutations in HSD11B2 and characterized by early-onset hypertension and hypokalemia. Endocrine [Internet]. [cited 2024 Oct 15];70:607–15. Available from: https://link.springer.com/10.1007/s12020-020-02460-9 10.1007/s12020-020-02460-9 PMC7674368 32816205

[191] Ceccato F, Mantero F (2019) Monogenic Forms of Hypertension. Endocrinol Metab Clin North Am [Internet]. [cited 2024 Oct 15];48:795–810. Available from: https://linkinghub.elsevier.com/retrieve/pii/S0889852919300635 10.1016/j.ecl.2019.08.009 31655777

[192] Tapia-Castillo A, Baudrand R, Vaidya A, Campino C, Allende F, Valdivia C et al (2019) Clinical, Biochemical, and Genetic Characteristics of Nonclassic Apparent Mineralocorticoid Excess Syndrome. J Clin Endocrinol Metab [Internet]. [cited 2024 Oct 15];104:595–603. Available from: https://academic.oup.com/jcem/article/104/2/595/5096788 10.1210/jc.2018-01197 30239803

[193] Razzaghy-Azar M, Yau M, Khattab A, New MI Apparent mineralocorticoid excess and the long term treatment of genetic hypertension. J Steroid Biochem Mol Biol [Internet]. 2017 [cited 2024 Oct 15];165:145–50. Available from: https://linkinghub.elsevier.com/retrieve/pii/S0960076016300280 10.1016/j.jsbmb.2016.02.014 26892095

[194] Vehaskari VM (2009) Heritable forms of hypertension. Pediatr Nephrol [Internet]. [cited 2024 Oct 15];24:1929–37. Available from: http://link.springer.com/10.1007/s00467-007-0537-8 10.1007/s00467-007-0537-8 PMC2755789 17647025

[195] Simonetti GD, Mohaupt MG, Bianchetti MG (2012) Monogenic forms of hypertension. Eur J Pediatr [Internet]. [cited 2024 Oct 15];171:1433–9. Available from: http://link.springer.com/10.1007/s00431-011-1440-7 10.1007/s00431-011-1440-7 21404100

[196] New MI, Geller DS, Fallo F, Wilson RC (2005) Monogenic low renin hypertension. Trends Endocrinol Metab [Internet]. [cited 2024 Oct 15];16:92–7. Available from: https://linkinghub.elsevier.com/retrieve/pii/S1043276005000408 10.1016/j.tem.2005.02.011 15808805

[197] Lu Y, Zhang D, Zhang Q, Zhou Z, Yang K, Zhou X et al (2022) Apparent mineralocorticoid excess: comprehensive overview of molecular genetics. J Transl Med [Internet]. [cited 2024 Oct 15];20:500. Available from: https://translational-medicine.biomedcentral.com/articles/10.1186/s12967-022-03698-9 10.1186/s12967-022-03698-9 PMC9632093 36329487

[198] Ferrari P The role of 11β-hydroxysteroid dehydrogenase type 2 in human hypertension. Biochim Biophys Acta BBA - Mol Basis Dis [Internet]. 2010 [cited 2024 Oct 15];1802:1178–87. Available from: https://linkinghub.elsevier.com/retrieve/pii/S0925443909002531 10.1016/j.bbadis.2009.10.017 19909806

[199] Kamide K, Kokubo Y, Hanada H, Nagura J, Yang J, Takiuchi S et al (2006) Genetic Variations of HSD11B2 in Hypertensive Patients and in the General Population, Six Rare Missense/Frameshift Mutations. Hypertens Res [Internet]. [cited 2024 Oct 15];29:243–52. Available from: http://www.nature.com/doifinder/10.1291/hypres.29.243 10.1291/hypres.29.243 16778331

[200] Carvajal CA, Tapia-Castillo A, Vecchiola A, Baudrand R, Fardella CE Classic and Nonclassic Apparent Mineralocorticoid Excess Syndrome. J Clin Endocrinol Metab [Internet]. 2020 [cited 2024 Oct 15];105:e924–36. Available from: https://academic.oup.com/jcem/article/105/4/e924/5691192 10.1210/clinem/dgz315 31909799

[201] Khandelwal P, Deinum J Monogenic forms of low-renin hypertension: clinical and molecular insights. Pediatr Nephrol [Internet]. 2022 [cited 2024 Oct 15];37:1495–509. Available from: https://link.springer.com/10.1007/s00467-021-05246-x 10.1007/s00467-021-05246-x 34414500

[202] Rossi GM, Regolisti G, Peyronel F, Fiaccadori E Recent insights into sodium and potassium handling by the aldosterone-sensitive distal nephron: a review of the relevant physiology. J Nephrol [Internet]. 2020 [cited 2024 Oct 15];33:431–45. Available from: http://link.springer.com/10.1007/s40620-019-00684-1 10.1007/s40620-019-00684-1 31950375

[203] Bubien JK (2010) Epithelial Na + Channel (ENaC), Hormones, and Hypertension. J Biol Chem [Internet]. [cited 2024 Oct 15];285:23527–31. Available from: https://linkinghub.elsevier.com/retrieve/pii/S0021925820618166 10.1074/jbc.R109.025049 PMC2911345 20460373

[204] Rotin D, Schild L ENaC and Its Regulatory Proteins as Drug Targets for Blood Pressure Control. Curr Drug Targets [Internet]. 2008 [cited 2024 Oct 15];9:709–16. 10.2174/138945008785132367 18691017

[205] Tetti M, Monticone S, Burrello J, Matarazzo P, Veglio F, Pasini B et al (2018) Liddle Syndrome: Review of the Literature and Description of a New Case. Int J Mol Sci [Internet]. [cited 2024 Oct 15];19:812. Available from: https://www.mdpi.com/1422-0067/19/3/812 10.3390/ijms19030812 PMC5877673 29534496

[206] Raina R, Krishnappa V, Das A, Amin H, Radhakrishnan Y, Nair NR et al (2019) Overview of Monogenic or Mendelian Forms of Hypertension. Front Pediatr [Internet]. [cited 2024 Oct 15];7:263. Available from: https://www.frontiersin.org/article/10.3389/fped.2019.00263/full 10.3389/fped.2019.00263 PMC6613461 31312622

[207] Liu K, Qin F, Sun X, Zhang Y, Wang J, Wu Y et al (2018) Analysis of the genes involved in Mendelian forms of low-renin hypertension in Chinese early-onset hypertensive patients. J Hypertens [Internet]. [cited 2024 Oct 15];36:502–9. Available from: https://journals.lww.com/00004872-201803000-00010 10.1097/HJH.0000000000001556 28915228

[208] Wang L-P, Yang K-Q, Jiang X-J, Wu H-Y, Zhang H-M, Zou Y-B et al (2015) Prevalence of liddle syndrome among young hypertension patients of undetermined cause in a Chinese population. J Clin Hypertens Greenwich Conn 17:902–907 10.1111/jch.12598 PMC8031848 26075967

[209] Martinez-Aguayo A, Fardella C (2009) Genetics of Hypertensive Syndrome. Horm Res Paediatr [Internet]. [cited 2024 Oct 15];71:253–9. Available from: https://karger.com/HRE/article/doi/10.1159/000208798 10.1159/000208798 19339789

[210] Cui Y, Tong A, Jiang J, Wang F, Li C Liddle syndrome: clinical and genetic profiles. J Clin Hypertens [Internet]. 2017 [cited 2024 Oct 15];19:524–9. Available from: https://onlinelibrary.wiley.com/doi/10.1111/jch.12949 10.1111/jch.12949 PMC8030933 27896928

[211] Salih M, Gautschi I, Van Bemmelen MX, Di Benedetto M, Brooks AS, Lugtenberg D et al (2017) A Missense Mutation in the Extracellular Domain of αENaC Causes Liddle Syndrome. J Am Soc Nephrol [Internet]. [cited 2024 Oct 15];28:3291–9. Available from: https://journals.lww.com/00001751-201711000-00020 10.1681/ASN.2016111163 PMC5661275 28710092

[212] Charoensri S, Auchus RJ (2023) Therapeutic management of congenital forms of endocrine hypertension. Eur J Endocrinol 189:R11–22 37847213 10.1093/ejendo/lvad140

[213] Fan P, Zhang D, Pan X-C, Yang K-Q, Zhang Q-Y, Lu Y-T et al Premature Stroke Secondary to Severe Hypertension Results from Liddle Syndrome Caused by a Novel SCNN1B Mutation. Kidney Blood Press Res [Internet]. 2020 [cited 2024 Oct 15];45:603–11. Available from: https://karger.com/KBR/article/doi/10.1159/000507580 10.1159/000507580 32698182

[214] Steyn N, Chale-Matsau B, Abera AB, Van Biljon G, Pillay TS (2023) Neonatal presentation of a patient with Liddle syndrome, South Africa. Afr J Lab Med [Internet]. [cited 2024 Oct 15];12. Available from: https://ajlmonline.org/index.php/ajlm/article/view/1998 10.4102/ajlm.v12i1.1998 PMC10157420 37151815

[215] Brower RK, Ghlichloo IA, Shabgahi V, Elsholz D, Menon RK, Vyas AK (2021) Liddle Syndrome due to a Novel c.1713 Deletion in the Epithelial Sodium Channel β-Subunit in a Normotensive Adolescent. AACE Clin Case Rep [Internet]. [cited 2024 Oct 15];7:65–8. Available from: https://linkinghub.elsevier.com/retrieve/pii/S237606052031018X 10.1016/j.aace.2020.11.017 PMC7924163 33851023

[216] O’Shaughnessy KM (2015) Gordon Syndrome: a continuing story. Pediatr Nephrol [Internet]. [cited 2024 Oct 15];30:1903–8. Available from: http://link.springer.com/10.1007/s00467-014-2956-7 10.1007/s00467-014-2956-7 25503323

[217] Hadchouel J, Delaloy C, Fauré S, Achard J-M, Jeunemaitre X Familial Hyperkalemic Hypertension. J Am Soc Nephrol [Internet]. 2006 [cited 2024 Oct 15];17:208–17. Available from: https://journals.lww.com/00001751-200601000-00028 10.1681/ASN.2005030314 16221868

[218] Costa-Barbosa FA, Giorgi RB, Kater CE Focus on adrenal and related causes of hypertension in childhood and adolescence: Rare or rarely recognized? Arch Endocrinol Metab [Internet]. 2022 [cited 2024 Oct 15]; Available from: https://www.aem-sbem.com/article/focus-on-adrenal-and-related-causes-of-hypertension-in-childhood-and-adolescence-rare-or-rarely-recognized/10.20945/2359-3997000000507 PMC10118774 35929903

[219] Pelham CJ, Ketsawatsomkron P, Groh S, Grobe JL, de Lange WJ, Ibeawuchi S-RC et al Cullin-3 Regulates Vascular Smooth Muscle Function and Arterial Blood Pressure via PPARγ and RhoA/Rho-Kinase. Cell Metab [Internet]. 2012 [cited 2024 Oct 15];16:462–72. Available from: https://linkinghub.elsevier.com/retrieve/pii/S1550413112003622 10.1016/j.cmet.2012.08.011 PMC3474846 23040068

[220] Boyden LM, Choi M, Choate KA, Nelson-Williams CJ, Farhi A, Toka HR et al (2012) Mutations in kelch-like 3 and cullin 3 cause hypertension and electrolyte abnormalities. Nature [Internet]. [cited 2024 Oct 15];482:98–102. Available from: https://www.nature.com/articles/nature10814 10.1038/nature10814 PMC3278668 22266938

[221] Gereda JE, Bonilla-Felix M, Kalil B, Dewitt SJ (1996) Neonatal presentation of Gordon syndrome. J Pediatr [Internet]. [cited 2024 Oct 15];129:615–7. Available from: https://linkinghub.elsevier.com/retrieve/pii/S0022347696701312 10.1016/s0022-3476(96)70131-2 8859273

[222] Mayan H, Munter G, Shaharabany M, Mouallem M, Pauzner R, Holtzman EJ et al (2004) Hypercalciuria in Familial Hyperkalemia and Hypertension Accompanies Hyperkalemia and Precedes Hypertension: Description of a Large Family with the Q565E WNK4 Mutation. J Clin Endocrinol Metab [Internet]. [cited 2024 Oct 15];89:4025–30. Available from: https://academic.oup.com/jcem/article-lookup/doi/10.1210/jc.2004-0037 10.1210/jc.2004-0037 15292344

[223] Park JH, Kim JH, Ahn YH, Kang HG, Ha IS, Cheong HI (2022) Gordon syndrome caused by a *CUL3* mutation in a patient with short stature in Korea: a case report. J Pediatr Endocrinol Metab [Internet]. [cited 2024 Oct 15];35:253–7. Available from: https://www.degruyter.com/document/doi/10.1515/jpem-2021-0361/html 10.1515/jpem-2021-0361 34480842

[224] Gordon RD (1986) Syndrome of hypertension and hyperkalemia with normal glomerular filtration rate. Hypertension [Internet]. [cited 2024 Oct 15];8:93–102. Available from: https://www.ahajournals.org/doi/10.1161/01.HYP.8.2.93 10.1161/01.hyp.8.2.93 3002982

[225] International Consortium for Blood Pressure (ICBP), Louis-Dit-Picard H, Barc J, Trujillano D, Miserey-Lenkei S, Bouatia-Naji N et al (2012) KLHL3 mutations cause familial hyperkalemic hypertension by impairing ion transport in the distal nephron. Nat Genet [Internet]. [cited 2024 Oct 15];44:456–60. Available from: https://www.nature.com/articles/ng.2218 10.1038/ng.2218 22406640

[226] Gordon RD, Hodsman GP (1986) The Syndrome of Hypertension and Hyperkalaemia without Renal Failure: Long Term Correction by Thiazide Diuretic. Scott Med J [Internet]. [cited 2024 Oct 15];31:43–4. Available from: https://journals.sagepub.com/doi/10.1177/003693308603100114 10.1177/003693308603100114 3961473

[227] Nicolaides NC, Charmandari E (2021) Primary Generalized Glucocorticoid Resistance and Hypersensitivity Syndromes: A 2021 Update. Int J Mol Sci [Internet]. [cited 2024 Oct 15];22:10839. Available from: https://www.mdpi.com/1422-0067/22/19/10839 10.3390/ijms221910839 PMC8509180 34639183

[228] Charmandari E, Kino T, Chrousos GP. Primary Generalized Familial and Sporadic Glucocorticoid Resistance (Chrousos Syndrome) and Hypersensitivity. In: Maghnie M, Loche S, Cappa M, Ghizzoni L, Lorini R,Endocr Dev [Internet]., Karger S (2013) AG; [cited 2024 Oct 15]. pp. 67–85. Available from: https://karger.com/books/book/207/chapter/5139153 10.1159/000342505 PMC4133123 23392096

[229] Vitellius G, Trabado S, Hoeffel C, Bouligand J, Bennet A, Castinetti F et al (2018) Significant prevalence of NR3C1 mutations in incidentally discovered bilateral adrenal hyperplasia: results of the French MUTA-GR Study. Eur J Endocrinol [Internet]. [cited 2024 Oct 15];178:411–23. Available from: https://academic.oup.com/ejendo/article/178/4/411/6655305 10.1530/EJE-17-1071 29444898

[230] Nicolaides N, Lamprokostopoulou A, Sertedaki A, Charmandari E Recent advances in the molecular mechanisms causing primary generalized glucocorticoid resistance. HORMONES [Internet]. 2016 [cited 2024 Oct 15]; Available from: http://www.hormones.gr/8621/article/recent-advances-in-the-molecular%E2%80%A6.html 10.14310/horm.2002.1660 27086682

[231] Nicolaides NC, Charmandari E (2015) Chrousos syndrome: from molecular pathogenesis to therapeutic management. Eur J Clin Invest [Internet]. [cited 2024 Oct 15];45:504–14. Available from: https://onlinelibrary.wiley.com/doi/10.1111/eci.12426 10.1111/eci.12426 25715669

[232] Koch C, Papadopoulou-Marketou N, Chrousos GP (2000) Overviewof endocrine hypertension. In: Feingold KR, Anawalt B,Blackman MR, Boyce A, Chrousos G, Corpas E et al (eds) Endotext.South Dartmouth (MA). MDText.com, Inc.

